# Fabrication of Ultrafine, Highly Ordered Nanostructures Using Carbohydrate-Inorganic Hybrid Block Copolymers

**DOI:** 10.3390/nano12101653

**Published:** 2022-05-12

**Authors:** Taiki Nishimura, Satoshi Katsuhara, Chaehun Lee, Brian J. Ree, Redouane Borsali, Takuya Yamamoto, Kenji Tajima, Toshifumi Satoh, Takuya Isono

**Affiliations:** 1 Graduate School of Chemical Sciences and Engineering, Hokkaido University, Sapporo 060-8628, Japan; taiki-nsm-1216@eis.hokudai.ac.jp (T.N.); satoshi-k@eis.hokudai.ac.jp (S.K.); dlcogns7942@eis.hokudai.ac.jp (C.L.); 2 Division of Applied Chemistry, Faculty of Engineering, Hokkaido University, Sapporo 060-8628, Japan; brianree@eng.hokudai.ac.jp (B.J.R.); yamamoto.t@eng.hokudai.ac.jp (T.Y.); ktajima@eng.hokudai.ac.jp (K.T.); 3 Centre de Recherches sur les Macromolécules Végétales (CERMAV), Centre National de la Recherche Scientifique (CNRS), Université Grenoble Alpes, F-38000 Grenoble, France; borsali@cermav.cnrs.fr

**Keywords:** organic–inorganic hybrid, block copolymer, self-assembly, microphase-separated structure, gyroid structure

## Abstract

Block copolymers (BCPs) have garnered considerable interest due to their ability to form microphase-separated structures suitable for nanofabrication. For these applications, it is critical to achieve both sufficient etch selectivity and a small domain size. To meet both requirements concurrently, we propose the use of oligosaccharide and oligodimethylsiloxane as hydrophilic and etch-resistant hydrophobic inorganic blocks, respectively, to build up a novel BCP system, i.e., carbohydrate-inorganic hybrid BCP. The carbohydrate-inorganic hybrid BCPs were synthesized via a click reaction between oligodimethylsiloxane with an azido group at each chain end and propargyl-functionalized maltooligosaccharide (consisting of one, two, and three glucose units). In the bulk state, small-angle X-ray scattering revealed that these BCPs microphase separated into gyroid, asymmetric lamellar, and symmetric lamellar structures with domain-spacing ranging from 5.0 to 5.9 nm depending on the volume fraction. Additionally, we investigated microphase-separated structures in the thin film state and discovered that the BCP with the most asymmetric composition formed an ultrafine and highly oriented gyroid structure as well as in the bulk state. After reactive ion etching, the gyroid thin film was transformed into a nanoporous-structured gyroid SiO_2_ material, demonstrating the material’s promising potential as nanotemplates.

## 1. Introduction

Organic–inorganic hybrid materials, which exhibit distinct properties due to the presence of both organic and inorganic components, have emerged as high-performance materials with a wide range of potential applications in a variety of fields [1,2,3,4,5]. Organic–inorganic hybrid block copolymers (BCPs) in particular have garnered considerable interest due to the fact that microphase separation enables the ordered periodic arrangement of organic and inorganic domains at the nanoscale [3,5,6,7]. Depending on the volume fraction of each block, it is possible to arrange not only alternating organic and inorganic layers in a lamellar (LAM) pattern but also in more complex patterns such as hexagonal cylinder, gyroid (GYR), and body-centered cubic sphere [8]. Because inorganic polymers are more resistant to etching and heat than organic polymers, organic–inorganic hybrid BCPs exhibit greater etching selectivity than conventional organic–organic BCPs [9]. Indeed, Zelsmann’s group [10], Fleury’s group [11], and Gopalan’s group [12] successfully synthesized poly(dimethylsiloxane)-*block*-polystyrene (PDMS-*b*-PS) and PDMS-*block*-poly(methyl methacrylate) (PDMS-*b*-PMMA) and transferred the patterns to the silicon substrate. Additionally, Cheng’s group [13] developed nanoporous materials with defined nanopore structures by selectively removing organic blocks from self-assembled polyhedral oligomeric silsesquioxane-*block*-PS (POSS-*b*-PS). The organic block decomposes into H_2_O and CO_2_, whereas the silicon-containing block converts to mechanically and thermally stable SiO_2_. Thus, these pioneering studies established the promising potential of combining organic and silicon-containing polymers for a variety of nanotemplate applications.

Controlling the domain spacing (*d*) of microphase-separated structures is critical for producing nanoobjects with desired dimensions when used as nanotemplates. However, the majority of organic–inorganic hybrid BCPs formed a microphase-separated structure with a *d* of at least 10 nm. Vantomme’s group [14] previously reported an organic–inorganic hybrid BCP that formed a microphase-separated structure with a *d* of 3.5 nm, but only the lamellar structure was observed. As a result, fabricating various nanostructures with a 5 nm scale periodicity remains a significant challenge for applications in nanotechnology, such as next-generation lithography, and the synthesis of a novel BCP capable of achieving microphase-separated structures with a *d* of around 5 nm or less is desired.

The *d* of the microphase-separated structure scales is described as *d*~*χ*^1/6^*N*^2/3^, where *χ* and *N* are the Flory–Huggins interaction parameter and degree of polymerization, respectively, implying that *d* can be decreased by lowering *N* [8]. However, because BCP is required to have an *χN* greater than a critical value (10.5 for symmetric diblock copolymer) in order to be microphase separated, simply decreasing *N* will result in a disordered state at some point. This is the fundamental reason for the difficulty in fabricating microphase-separated structures with *d* less than 10 nm [8]. To overcome this constraint, the enhancement of *χ* is required, and indeed, numerous research groups have demonstrated microphase-separation from so-called “high *χ*/low *N*” BCPs, resulting in ordered morphologies with *d* of less than 10 nm. Thus far, silicon-containing [3,5,6,7,10,12,14,15,16,17,18,19,20,21,22,23,24,25,26,27,28,29,30], metal-doped [31,32,33], and oligosaccharide-based BCPs [34,35,36,37,38,39,40,41,42,43,44,45,46,47,48] have been developed. The high *χ*/low *N* BCP strategy is now widely accepted as a standard method for obtaining smaller *d*, although several novel approaches have emerged recently [19,34,35,40,49,50,51,52,53,54,55,56,57].

When considering organic–inorganic hybrid BCPs for nanotemplate applications, the interfacial width between the two phases should be minimized, as a wider interface results in defect formation and an increase in line edge roughness. Solving these issues is critical for nanofabrication in a sub-10 nm scale. As the interfacial width is proportional to *χ*^−1/2^, increasing *χ* is also critical for successful nanofabrication [8]. Thus, maximizing *χ* is an extraordinary challenge not only for achieving microphase separation in the low molecular weight region but also for precise nanofabrication.

In general, as the molecular weight of low *N* BCPs used to achieve ultrafine *d* decreases, the mechanical and thermal properties reduce significantly. This could have a detrimental effect on the performance of organic–inorganic hybrid BCP as a nanotemplate material. The use of triblock copolymer architecture may provide a solution to this problem. When *N*_ABA_ = *N*_AB_, the microphase-separated morphology and dimension of ABA-type triblock copolymers are comparable to those of AB-type diblock copolymers. In other words, *d* of an ABA-type triblock copolymer is half that of an AB-type diblock copolymer with the same molecular weight [58]. Thus, using the organic–inorganic hybrid triblock copolymer architecture would be extremely advantageous for forming ultrafine microphase-separated structures without sacrificing the molecular weight of BCP.

**Scheme 1:**
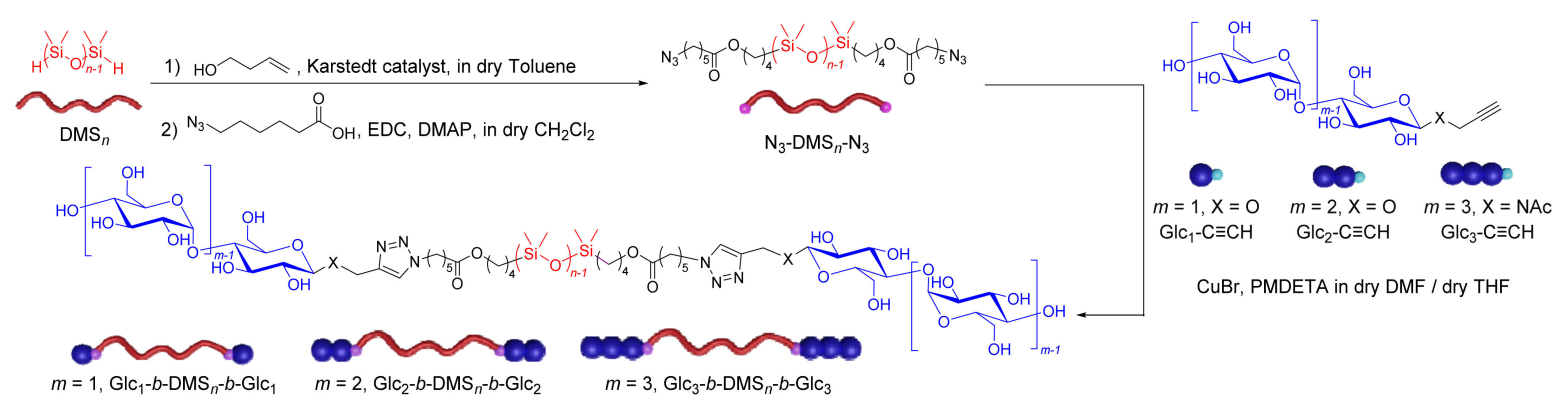
Synthesis of Glc*_m_*-*b*-DMS*_n_*-*b*-Glc*_m_* (*m* = 1–3).

Herein, we present an ultrahigh *χ*/low *N* organic-inorganic hybrid BCP system with an ABA-type triblock architecture that exhibits ultrafine microphase separation in the bulk and thin film states, indicating potential nanofabrication applications. The majority of previously reported organic–inorganic hybrid BCPs were composed of hydrophobic organic and inorganic polymers, such as PS/PDMS and polylactide/PDMS. However, it is reasonable to conclude that BCPs composed of hydrophilic organic polymers and hydrophobic inorganic polymers achieve significantly greater *χ*. When comparing the solubility parameter table of common polymers, PDMS is ranked as one of the lowest and polysaccharides are ranked as the highest. The Flory–Huggins theory states that the greater the difference in the solubility parameter, the greater *χ* is. Thus, we examined the combination of carbohydrate and PDMS as a hydrophilic organic polymer and a hydrophobic inorganic polymer, respectively, in this study. More precisely, we used maltooligosaccharides as the carbohydrate building block because maltooligosaccharides with a discrete number of glucose repeating units are readily available, allowing for the precise control of the volume fraction and, consequently, the resulting morphology. Indeed, our research group [37] and Sita’s research group [36] independently reported a variety of microphase-separated nanostructures derived from oligosaccharide-based organic BCPs by varying glucose unit counts. The molecular structures and synthetic route for the target ultrahigh *χ*/low *N* organic–inorganic hybrid triblock copolymers (Glc*_m_*-*b*-DMS*_n_*-*b*-Glc*_m_*; *m* = 1–3), which are composed of maltooligosaccharides (glucose, Glc_1_; maltose, Glc_2_; maltotriose, Glc_3_) and oligodimethylsiloxane (*o*DMS), are shown in Scheme 1. As this BCP system is a novel organic–inorganic hybrid BCP, it may be referred to as a “carbohydrate-inorganic hybrid BCP”. Despite having a molecular weight of less than 2500, Glc*_m_*-*b*-DMS*_n_*-*b*-Glc*_m_* self-assembled into highly ordered lamellar and gyroid morphologies in the bulk and thin film states. Owing to the ultrahigh *χ*/low *N* nature of the material and the ABA-type architecture, a *d* of as little as 5 nm was achieved, which is among the smallest in microphase-separated organic–inorganic hybrid BCPs [14,25,29,30].

## 2. Results and Discussion

### 2.1. Synthesis

**Figure 1:**
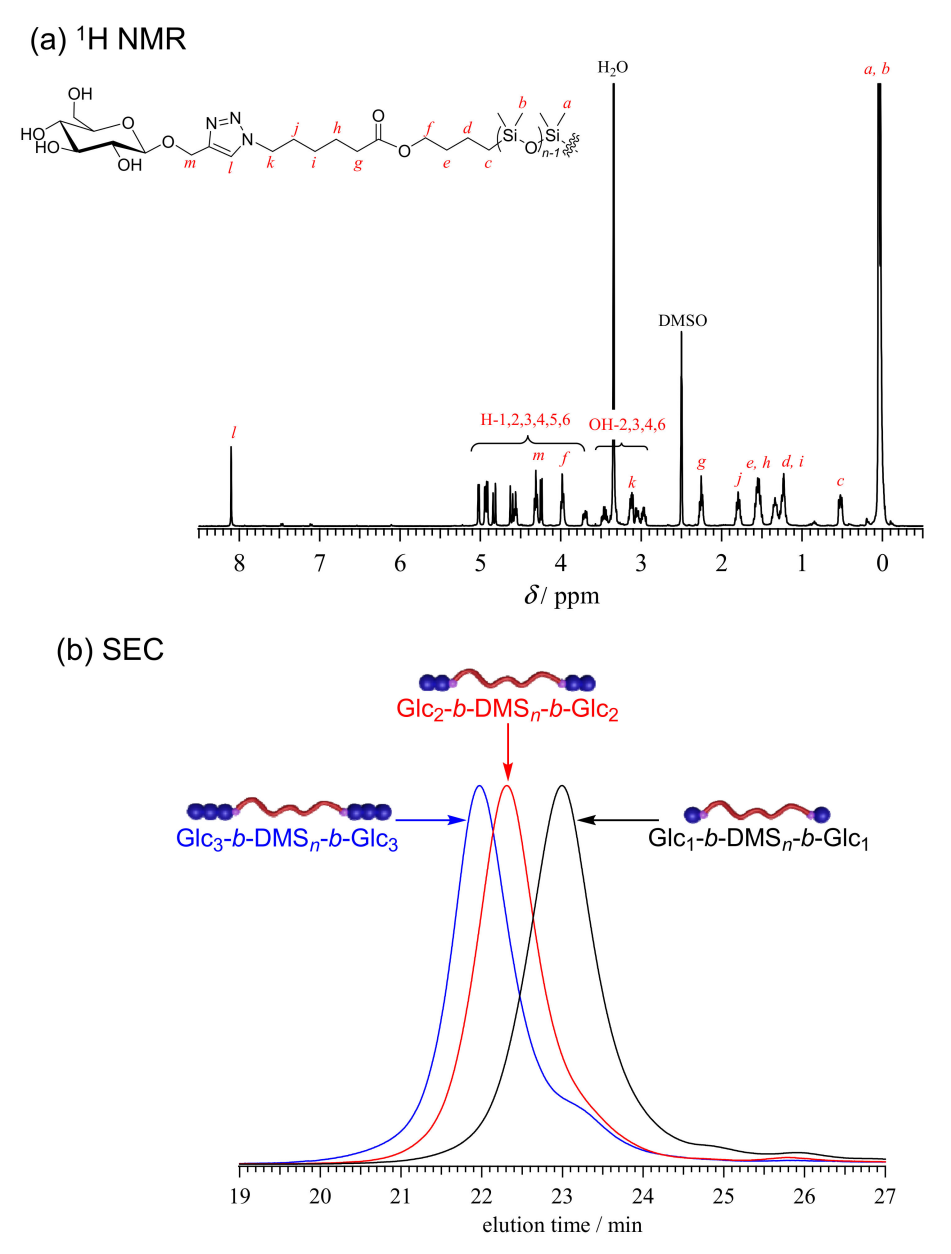
(**a**) ^1^H NMR spectrum of Glc_1_-*b*-DMS*_n_*-*b*-Glc_1_ in DMSO-*d*_6_ (400 MHz); (**b**) SEC traces of Glc_1_-*b*-DMS*_n_*-*b*-Glc_1_ (black), Glc_2_-*b*-DMS*_n_*-*b*-Glc_2_ (red) and Glc_3_-*b*-DMS*_n_*-*b*-Glc_3_ (blue) (eluent, DMF containing 0.01 mol L^−1^ LiCl; flow rate, 0.60 mL min^−1^).

Our BCPs (Glc*_m_*-*b*-DMS*_n_*-*b*-Glc*_m_*; *m* = 1–3) were synthesized via click reaction between azido-modified *o*DMS (N_3_–DMS*_n_*–N_3_) and propargyl-modified sugar units (Glc*_m_*–C≡CH) (Figures S1–S3). The synthesis began with hydrosilylation of 3-buten-1-ol with H–DMS*_n_*–H in dry toluene in the presence of a Karstedt catalyst, yielding HO–DMS*_n_*–OH in 68.9%. The chain end hydroxyl groups were then reacted with 6-azidohexanoic acid in the presence of 1-ethyl-3-(3-dimethylaminopropyl)carbodiimide hydrochloride (EDC) and 4-dimethylaminopyridine (DMAP) to produce an *α*,*ω*-diazido-functionalized *o*DMS (N_3_–DMS*_n_*–N_3_). The azido group was successfully introduced as evidenced by ^1^H NMR analysis, in which the methylene proton adjacent to the hydroxyl group vanished completely (Figures S4 and S5). The click reactions of N_3_–DMS*_n_*–N_3_ with Glc*_m_*–C≡CH (*m* = 1–3) were carried out at 60 °C in a mixture of dry *N*,*N*-dimethylformamide (dry DMF) and dry tetrahydrofuran (dry THF) (1/1 (*v*/*v*)), in the presence of copper(I) bromide (CuBr) and *N*, *N*, *N′*, *N″*, *N″*-pentamethyldiethlenetriamine (PMDETA) as a catalyst and a ligand, respectively. Following confirmation of complete azido group consumption via FT-IR analysis, the desired BCPs, viz., Glc*_m_*-*b*-DMS*_n_*-*b*-Glc*_m_* (*m* = 1–3), were obtained in a yield of 23.6–40.3% following treatment with the cation exchange resin followed by reprecipitation in methanol to remove catalyst residues and excess Glc*_m_*–C≡CH (Table 1 and Figure S6). Size-exclusion chromatography (SEC), FT-IR, and ^1^H NMR analyses confirmed the successful synthesis (Figure 1 and Figures S7–S14). For each BCP, a monomodal SEC elution peak was observed on the higher molecular-weight side of the reactants while maintaining a narrow molecular weight distribution (*Ð* = 1.08–1.12) (Figure 1b and Figures S7–S9). No characteristic absorption band due to the azido group at around 2100 cm^−1^ was observed in the FT-IR spectra of BCPs, indicating that the click reaction proceeded quantitatively (Figure S6). Both *o*DMS and oligosaccharide segments were reasonably attributed to the ^1^H NMR signals (Figure 1a, Figures S11 and S13). More importantly, at 8.10 ppm, a signal due to the methin proton in the newly formed triazole ring was observed, conforming the successful click reaction once again. The molecular weight (*M*_n,NMR_) and *o*DMS volume fraction (*f*_DMS*n*_) calculated using ^1^H NMR were listed in Table 1. To confirm the chemical structure of BCPs and to gain insight into their molecular weight distributions, electrospray ionization mass spectrometry (ESI-MS) analysis was performed. The ESI mass spectrum of Glc_1_-*b*-DMS*_n_*-*b*-Glc_1_ revealed multiple series of repeating peaks separated by approximately 74 Da in the 1300–2000 Da range, confirming the molecular weight distribution due to the dimethylsiloxane unit (74.1 Da) (Figure S16). All peaks were assigned to the same chemical species of Glc_1_-*b*-DMS*_n_*-*b*-Glc_1_ with varying cationizing agents (H^+^, Na^+^, Na^+^ + MeOH adducts). The peak at *m*/*z* = 1385.56, for example, corresponds to the theoretical molecular mass of the Na^+^ adduct of the 7-mer of Glc_1_-*b*-DMS*_n_*-*b*-Glc_1_ ([M + Na]^+^ = 1385.56 Da). These results indicate that Glc_1_-*b*-DMS*_n_*-*b*-Glc_1_ does not have a distribution of saccharide units but does have a distribution of dimethylsiloxane units ranging from 7 to 14. The same conclusion was also reached when Glc_2_-*b*-DMS*_n_*-*b*-Glc_2_ and Glc_3_-*b*-DMS*_n_*-*b*-Glc_3_ were analyzed using ESI-MS (Figures S17 and S18). These finding established the feasibility of synthesizing a series of BCPs with defined structures.

### 2.2. Thermal Properties

Thermogravimetric analysis (TGA) and differential scanning calorimetry (DSC) were used to characterize the thermal properties of the synthesized BCPs and their corresponding constituents. TGA was initially used to investigate the BCPs’ 5% weight loss temperatures (*T*_d_). Regardless of the number of glucose units in the BCPs, the TGA profiles demonstrated a two-step weight loss due to *o*DMS and saccharide segments (Figures S19–S22). By examining their weight fractions, the first degradation can be attributed to the saccharide segment, while the second can be attributed to the *o*DMS segment. In comparison, the *T*_d_ of Glc*_m_*–C≡CH (*m* = 1: 216.0 °C, *m* = 2: 214.8 °C, *m* = 3: 145.3 °C) is higher than that of N_3_–DMS*_n_*–N_3_ (136.9 °C). This observation may be explained by the fact that the thermal decomposition mechanism of *o*DMS alone differs from that of the BCP. Previous research has established that the depolymerization of PDMS is primarily governed by molecular structure and kinetics rather than by bond energies [56]. Additionally, it has been established that PDMS thermally decomposes via intramolecular siloxane bond rearrangement, resulting in the formation of smaller cyclic products, such as hexamethylcyclotrisiloxane. Taking these facts into account, the intramolecular siloxane bond rearrangement reaction of the *o*DMS block in BCPs may be suppressed by the presence of the bulky microphase-separated saccharide domain, resulting in an increase in the *T*_d_ of the *o*DMS block.

Following that, we compared the synthesized BCPs’ second heating DSC curves to their corresponding Glc*_m_*–C≡CH (Figures S23–S25) to determine their glass transition temperature (*T*_g_). The DSC curve of Glc_1_-*b*-DMS*_n_*-*b*-Glc_1_ demonstrated a baseline shift at −5.7 °C due to the glucose segment’s *T_g_*, whereas that of Glc_1_–C≡CH demonstrated a baseline shift at 38.3 °C. Glc_2_-*b*-DMS*_n_*-*b*-Glc_2_ also had a lower *T*_g_ (−0.9 °C) due to the maltose segment than Glc_2_–C≡CH (66.9 °C). The decrease in the saccharide segment’s *T*_g_ upon BCP formation could be explained by the segment’s partial miscibility with the *o*DMS segment due to its low *N*. In comparison, the *T*_g_ of the maltotriose segment in Glc_3_-*b*-DMS*_n_*-*b*-Glc_3_ (81.7 °C) was similar to that of Glc_3_–C≡CH (74.4 °C). Thus, an increase in *N* increases the immiscibility of the oligosaccharide and *o*DMS segments, which may account for the absence of partial miscibility in Glc_3_-*b*-DMS*_n_*-*b*-Glc_3_. Notably, no crystallization/melting was observed in the DSC scan range (−120–120 °C) due to the *o*DMS segments in BCPs. Additionally, no clear *T*_g_ for the *o*DMS segments was observed.

### 2.3. Microphase Separation Behavior in the Bulk State

The microphase-separated structures of the BCPs (Glc*_m_*-*b*-DMS*_n_*-*b*-Glc*_m_*; *m* = 1–3) in their bulk states were initially investigated using the wide- and small-angle X-ray scattering (WAXS and SAXS, respectively). All samples were thermally annealed at 80 and130 °C for 6 h and then rapidly quenched, followed by the acquisition of their WAXS/SAXS profiles at ambient temperature. All BCP samples had a single broad halo peak on their WAXS profiles, confirming their amorphous nature (Figure S26). This corresponds to the DSC results. SAXS profiles of 80 °C-annealed Glc_1_-*b*-DMS*_n_*-*b*-Glc_1_ revealed scattering peaks at *q* = 1.26, 1.45, 1.92, 2.30, 2.41, 2.52, 2.62, 2.81, 3.17, 3.33, 3.56, and 3.63 nm^−^^1^, which correspond to the crystallographic planes (211), (022), (123), (024), (233), (422), (134), (512), (145), (613), (444), and (543), respectively. Notably, Glc_1_-*b*-DMS*_n_*-*b*-Glc_1_ exhibited twelve scattering peaks, indicating that a highly ordered GYR structure was formed (Figure S27). Notably, even without thermal annealing, the clear GYR structure was discovered. The cubic lattice parameter *a*_cub_ was determined to be 12.3 nm regardless of the annealing condition using the slope of a plot of *q_hkl_* versus (*h*^2^ + *k*^2^ + *l*^2^)^1/2^ [*a*_cub_ = 2π(*h*^2^ + *k*^2^ + *l*^2^)^1/2^/*q_hkl_*], where *h*, *k*, and *l* are Miller indices (Figure S28). The *d* of (211) crystallographic plane of the GYR structure was also calculated using Bragg’s equation (*d* = 2π/q *), and it was determined to be 5.0 nm.

The formation of GYR in Glc_1_-*b*-DMS*_n_*-*b*-Glc_1_ appears to be quite unusual. GYR is typically formed from BCPs with a volume fraction of 0.35–0.39, but Glc_1_-*b*-DMS*_n_*-*b*-Glc_1_ lies outside this range (saccharide volume fraction is around 0.18). However, our previous study [37] demonstrated that when a saccharide volume fraction of 0.21–0.25 occurs, discrete BCPs composed of oligosaccharide and terpenoid blocks self-assembled into GYR. Additionally, Sita’s group [36] demonstrated that BCPs containing atactic polypropylene and cellobiose with a saccharide volume fraction of 0.23 formed GYR structures, which is consistent with our previous work [37]. Thus, the glucose block’s rigidity and strong intermolecular interaction may play a significant role in the formation of unusual GYR.

**Figure 2:**
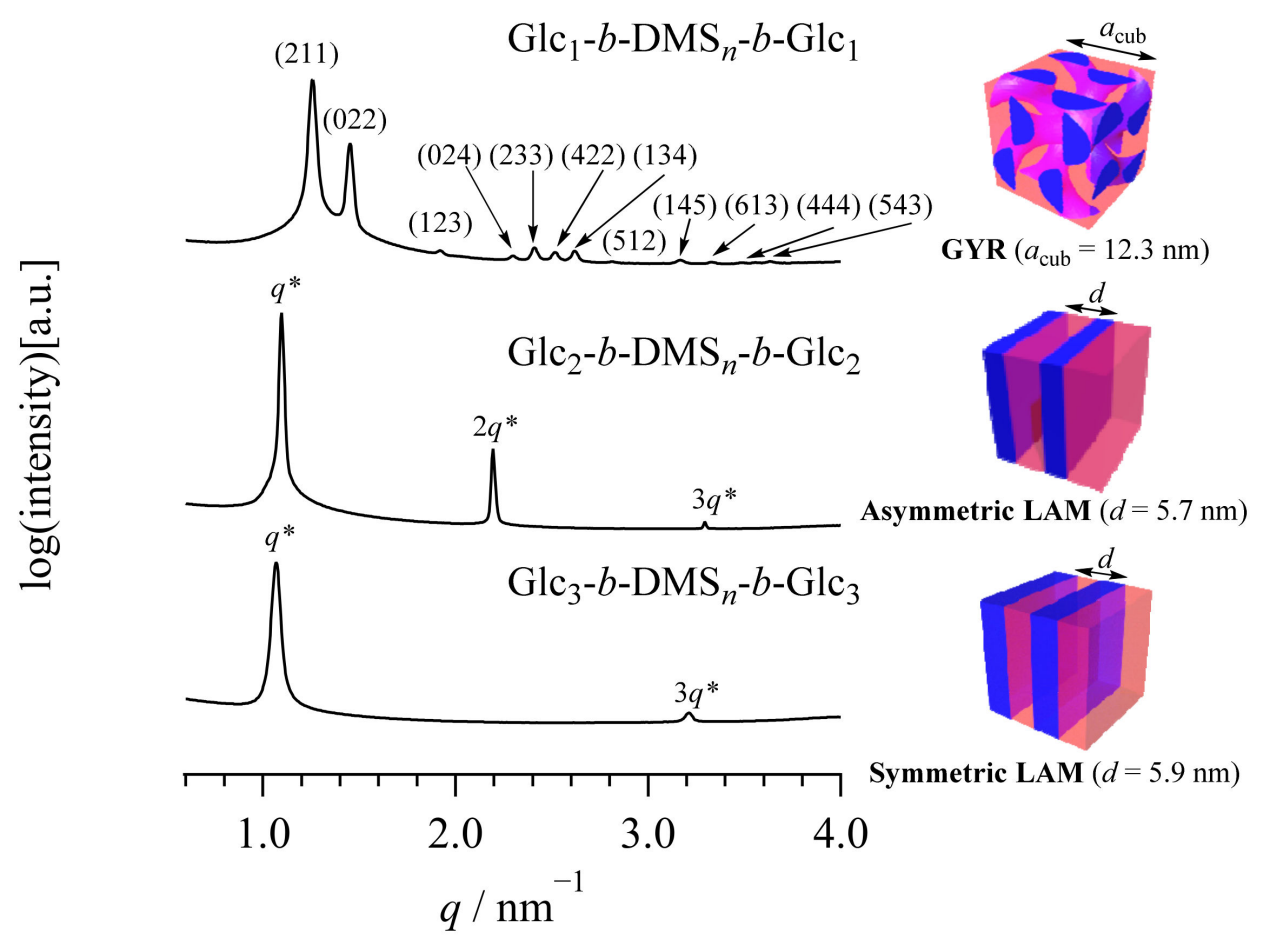
SAXS profiles of Glc_1_-*b*-DMS*_n_*-*b*-Glc_1_ (**upper**), Glc_2_-*b*-DMS*_n_*-*b*-Glc_2_ (**middle**), and Glc_3_-*b*-DMS*_n_*-*b*-Glc_3_ (**lower**). All samples were annealed at 80 °C for 6 h. The cubic lattice parameter *a*_cub_ for the GYR was calculated based on *a*_cub_ = 2π(*h*^2^ + *k*^2^ + *l*^2^)^1/2^/*q_hkl_* and the *d* value for the LAM was calculated based on Bragg’s equation: *d* = 2π/q *.

The LAM structures with domain-spacings (*d*) of 5.6–5.9 nm were discovered in Glc_2_-*b*-DMS*_n_*-*b*-Glc_2_ and Glc_3_-*b*-DMS*_n_*-*b*-Glc_3_ (Figures S29 and S30), as indicated by the higher ordered scattering peaks at the integer multiple positions (2q * and 3q *) relative to the primary peak. Notably, the even-ordered peak (2q *) was absent (or suppressed) in the SAXS profiles of Glc_3_-*b*-DMS*_n_*-*b*-Glc_3_ (Figure 2). This indicates that the two phases of the LAM structure were of equal thickness [59], corroborating the formation of a symmetric LAM structure with a domain thickness of 3 nm.

It was found that the increase in the saccharide volume fraction resulted in a shift of the resulting morphology from GYR to less curved LAM structures. Such morphology change relating to the volume fraction is commonly observed in conventional BCPs, in which the area-minimization of the intermaterial surface is an essential factor that determines the microphase-separated morphology at each volume fraction [60].

According to previous reports, low *N* diblock copolymers with a molecular weight of 1500–2500 tend to form microphase-separated structures with a *d* of 10–12 nm [22]. Contrastingly, our ABA-type triblock copolymers formed microphase-separated structures with *d* values of 5–6 nm, which is nearly half that of a diblock copolymer of comparable molecular weight. This validated the promising potential of an ABA-type triblock architecture for reducing the size of *d* in low *N* saccharide-based BCPs.

### 2.4. Microphase Separation Behavior in the Thin Film State

**Figure 3:**
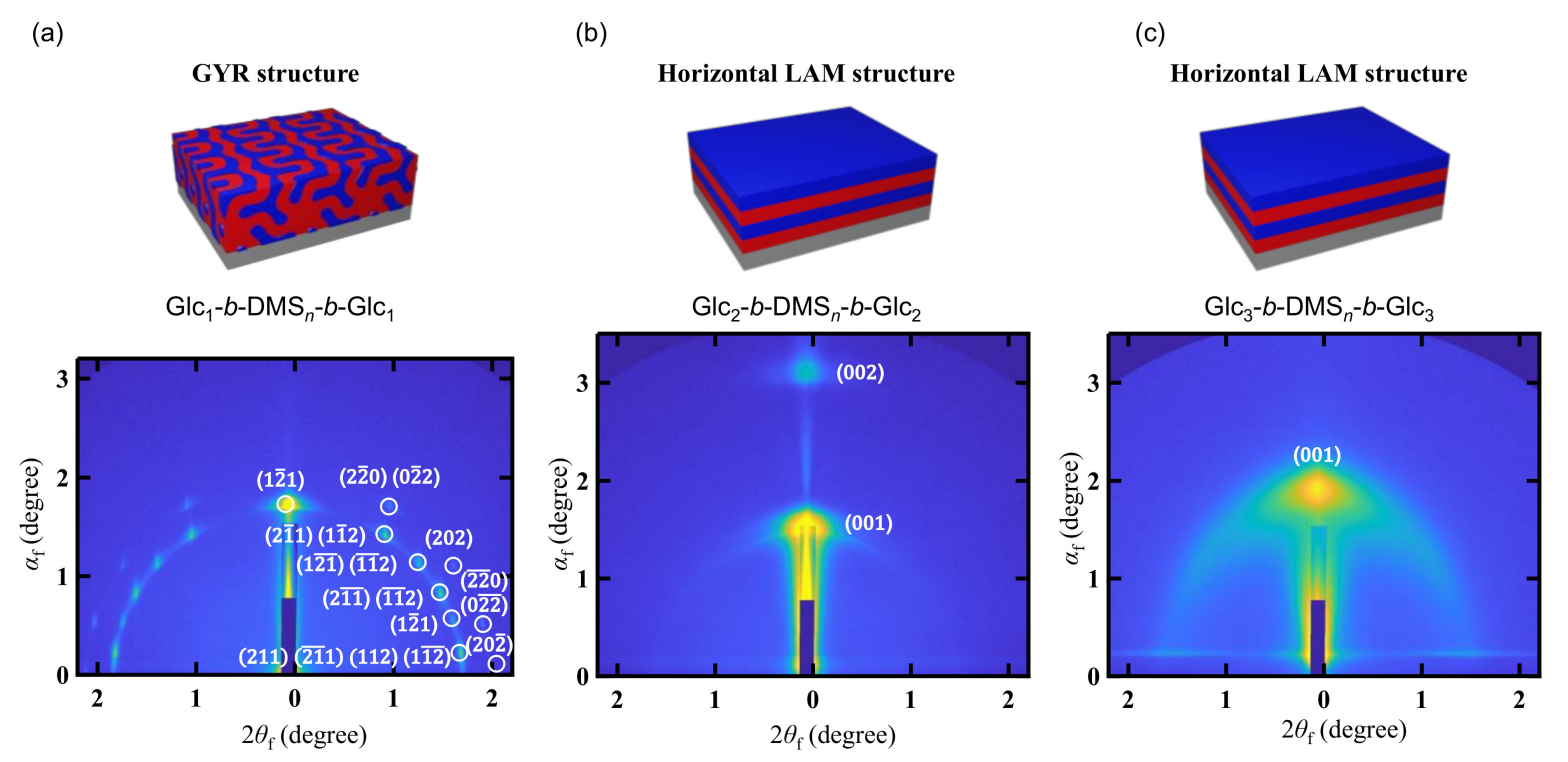
GISAXS images of (**a**) Glc_1_-*b*-DMS*_n_*-*b*-Glc_1_, (**b**) Glc_2_-*b*-DMS*_n_*-*b*-Glc_2_, and (**c**) Glc_3_-*b*-DMS*_n_*-*b*-Glc_3_ thin films. All samples were prepared by spin coating 5.0 wt% DMF solutions onto silicon substates and subsequent thermal annealed at 80 °C for 6 h.

The microphase-separated nanostructure was then investigated in the thin film state. The thin films were formed by spin coating 5.0 wt% DMF solutions onto silicon substrates and thermal annealing at 80 °C and 130 °C for 6 h. To begin, grazing incidence small-angle X-ray scattering (GISAXS) experiments were performed on Glc*_m_*-*b*-DMS*_n_*-*b*-Glc*_m_* (*m* = 1–3) thin films (Figure 3). The GISAXS images of Glc_1_-*b*-DMS*_n_*-*b*-Glc_1_ thin films revealed distinct scattering spots, which remained consistent regardless of the thermal annealing condition (Figure 3a and Figure S31). Each scattering spot was successfully assigned to the GYR structure. It is well established that GYR thin films exhibit a scattering pattern that varies depending on their orientation. Glc_1_-*b*-DMS*_n_*-*b*-Glc_1_ formed a uniformly oriented GYR structure inside the thin film, with the (121) plane parallel to silicon substrates [61] (Figure 3a). GISAXS analysis determined *a*_cub_ to be 12.5 nm, which is consistent with the result of bulk SAXS analysis.

To demonstrate the stability of the GYR structure, we performed GISAXS measurement on an eight-month-old Glc_1_-*b*-DMS*_n_*-*b*-Glc_1_ thin film (Figure S32). Surprisingly, the GISAXS images obtained immediately after preparation are qualitatively comparable to those obtained eight months later. This demonstrated that GYR’s morphology, dimension, and orientation remained stable over an eight-month period. This established that the observed GYR structure is most thermodynamically stable phase in the thin film state.

Regardless of the thermal annealing condition, the GISAXS images of Glc_2_-*b*-DMS*_n_*-*b*-Glc_2_ and Glc_3_-*b*-DMS*_n_*-*b*-Glc_3_ revealed two scattering spots along the out-of-plane direction (Figure 3b,c, see also Figures S33 and S34). This demonstrated the formation of parallel-oriented LAM structures on the silicon substrate (horizontal LAM structure).

When *d* values calculated from thin film GISAXS and bulk SAXS measurements were compared, it was discovered that Glc_2_-*b*-DMS*_n_*-*b*-Glc_2_ has *d* values of 5.7 nm in both thin film and bulk states. However, using SAXS and GISAXS, the *d* values of Glc_3_-*b*-DMS*_n_*-*b*-Glc_3_ were calculated to be 5.9 and 4.5 nm, respectively. We do not have a clear explanation for this contradiction at the moment. A possible explanation for the discrepancy with the bulk state is the geometrical confinement effect [61], which results in a different molecular packing of the saccharide block and a different proportion of loop/bridge conformation in the *o*DMS block than in the bulk state.

Following that, atomic force microscopy (AFM) was used to visualize the surface morphology of the Glc*_m_*-*b*-DMS*_n_*-*b*-Glc*_m_* (*m* = 1–3) thin films. Two distinct types of periodic nanostructures are visible in the AFM phase images of the 80 °C annealed Glc_1_-*b*-DMS*_n_*-*b*-Glc_1_ thin film. The first is composed of spherical dots arranged in a straight line, while the second is made up of spherical dots arranged in hexagonal patterns. By examining the morphology determined by SAXS/GISAXS (i.e., GYR), it is possible to attribute the former and later to the projections of the GYR (110) and (111) planes, respectively (Figures S35 and S36).

The AFM height images of the thin films prepared with Glc_2_-*b*-DMS*_n_*-*b*-Glc_2_ (annealed at 80 °C) and Glc_3_-*b*-DMS*_n_*-*b*-Glc_3_ (annealed at 80 °C) clearly demonstrated flat layers with a constant height difference between consecutive layers, which can be attributed to the horizontal LAM morphology. The height differences between the two thin films were in the range of 4–6 nm, which corresponded well to the *d* value determined by SAXS/GISAXS analysis (Figures S37–S42).

### 2.5. Preparation of GYR Structure Nanoporous Materials

Finally, we attempted to prepare a nanoporous material by utilizing the GYR nanostructure in the Glc_1_-*b*-DMS*_n_*-*b*-Glc_1_ thin film. The removal of the saccharide domain from the GYR structure should result in the formation of nanoporous membranes. To convert *o*DMS to SiO_2_ and selectively remove the saccharide block, three different reactive ion etching (RIE) processes were used, and each used a different gas flow rate or time. The first procedure used a 15 s O_2_ plasma etching (15 sccm) with a 250 W source power. The second procedure was carried out using a 35 s Ar/O_2_ plasma ecthing (80/40 sccm) powered by a 200 W source power. Finally, a 35 s long CF_4_/O_2_ plasma etching (60/60 sccm) was performed with a 250 W source power (a detailed description of RIE is provided in the SI).

**Figure 4:**
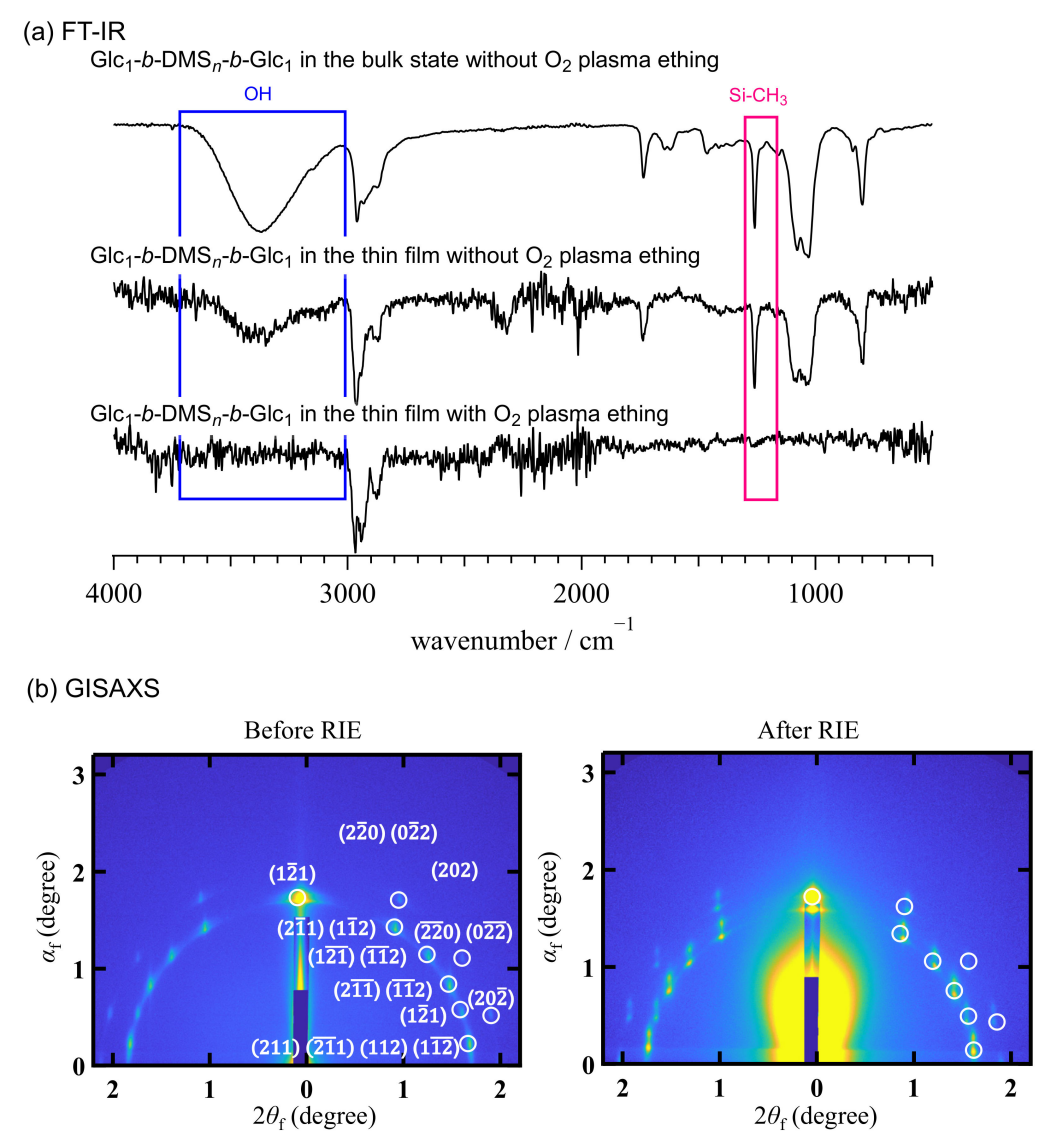
(**a**) ATR FT-IR spectra of Glc_1_-*b*-DMS*_n_*-*b*-Glc_1_ measured in the bulk state (**upper**) and measured in the thin film state before (**middle**) and after the RIE (**lower**); (**b**) GISAXS images of Glc_1_-*b*-DMS*_n_*-*b*-Glc_1_ before (**left**) and after the RIE (**right**).

In the ATR FT-IR spectrum prior to RIE, the Glc_1_-*b*-DMS*_n_*-*b*-Glc_1_ thin film exhibited characteristic broad absorption bands due to the hydroxyl group at approximately 3000 cm^−^^1^ and Si–CH_3_ at approximately 1250 cm^−^^1^, but these absorption bands vanished after RIE (Figure 4a). Similarly, the Raman spectrum of Glc_1_-*b*-DMS*_n_*-*b*-Glc_1_ thin film after RIE did not exhibit any peak at approximately 3000 cm^−^^1^ region due to the hydroxyl group (Figure S43). This confirmed that the saccharide block had been successfully removed. To obtain additional insight into the chemical characteristic change caused by RIE, we measured the water contact angle (WCA) of the Glc_1_-*b*-DMS*_n_*-*b*-Glc_1_ thin films before and after RIE (Figure S44). Before RIE, the WCA of Glc_1_-*b*-DMS*_n_*-*b*-Glc_1_ thin film was determined to be 99.1°, which was consistent with that of pure PDMS (99.8°) [62]. This indicates that *o*DMS covers the top surface of the thin film due to its lower surface free energy. After RIE, the WCA of the Glc_1_-*b*-DMS*_n_*-*b*-Glc_1_ thin film was determined to be 83.9°, which was in close agreement with that of SiO_2_ (87.5°) [63]. These findings indicated that the saccharide domain was removed selectively and the *o*DMS segment was converted to SiO_2_. Following that, we used GISAXS and AFM to investigate the nanostructural changes caused by RIE. Surprisingly, the GISAXS images of the thin film following RIE revealed a distinct pattern similar to that observed prior to the RIE. This demonstrated that the GYR structure, in which the (121) plane is parallel to the silicon substrates, was maintained throughout the etching process (Figure 4b). Additionally, even after RIE, the AFM image barely revealed an ordered nanopattern (Figure S45). Collectively, these findings indicated that a GYR-structured nanoporous SiO_2_ material could be synthesized by simply applying the RIE process to a BCP thin film.

## 3. Conclusions

Using the click reaction, we successfully synthesized ABA-type triblock copolymers composed of oligosaccharide and oligodimethylsiloxane segments (Glc*_m_*-*b*-DMS*_n_*-*b*-Glc*_m_*; *m* = 1–3). Self-assembly of the resulting BCPs produced well-ordered GYR, asymmetric LAM, and symmetric LAM nanostructures with an ultrafine *d* value (5.0–5.9 nm). Owing to the difficulty of obtaining such small *d* using diblock copolymers of comparable molecular weight, we established the advantage of the ABA-type triblock architecture for achieving ultrafine microphase-separated structures. Furthermore, the Glc_1_-*b*-DMS*_n_*-*b*-Glc_1_ thin film formed a stable and uniformly oriented GYR structure, which has potential as nanotemplates or precursors for nanoporous materials. Indeed, reactive ion etching of the Glc_1_-*b*-DMS*_n_*-*b*-Glc_1_ thin film resulted in the formation of a nanoporous SiO_2_ material with a gyroid structure. This demonstrated the benefit of combining inorganic polymers and saccharide blocks. Overall, it was determined that the combination of saccharide and *o*DMS in the triblock architecture is extremely promising for achieving ultrafine *d* and etching selectivity simultaneously. Given the ultrafine *d* and sufficient etching selectivity, our BCP is likely to find applications in nanolithographic templates, as precursors for nanoporous materials, in the fabrication of nanofiltration membranes and so on.

## Figures and Tables

**Table 1:** Molecular characteristics of Glc*_m_*-*b*-DMS*_n_*-*b*-Glc*_m_* (*m* = 1–3).

Sample	*M*_n,NMR_ *^a^*	*Ð ^b^*	*f*_DMS*n*_ *^c^*	Bulk State Morphology		
				Annealing Condition	Morphology	*D ^d^*/nm
Glc_1_-*b*-DMS*_n_*-*b*-Glc_1_	1610	1.08	0.82	non	GYR	5.0 *^e^*
				80 °C, 6 h	GYR	5.0 *^e^*
				130 °C, 6 h	GYR	5.0 *^e^*
Glc_2_-*b*-DMS*_n_*-*b*-Glc_2_	1950	1.08	0.73	non	LAM	5.6
				80 °C, 6 h	LAM	5.7
				130 °C, 6 h	LAM	5.7
Glc_3_-*b*-DMS*_n_*-*b*-Glc_3_	2350	1.12	0.62	non	LAM	5.9
				80 °C, 6 h	LAM	5.9
				130 °C, 6 h	LAM	5.9

*^a^* Determined by ^1^H NMR. *^b^* Determined by SEC in DMF using PMMA as the standard. *^c^* Calculated based on the density value for each block: 0.91 g cm^−3^ for DMS*_n_* block, 1.54 g cm^−3^ for Glc_1_ and Glc_2_ block, and 1.44 g cm^−3^ for Glc_3_ block. *^d^* Calculated based on Bragg’s equation: *d* = 2π/q * (q * is the position of the primary peak). *^e^ d* of (211) crystallographic plane of the GYR structure.

## Data Availability

The data presented in this study are available upon request from the corresponding author.

## Supplementary Materials

The following supporting information can be downloaded at https://www.mdpi.com/article/10.3390/nano12101653/s1, Experimental section, additional discussion for polymer syntheses, and ^1^H NMR, SEC, FT-IR, MALDI-TOF MS, TGA, DSC, WAXS, SAXS, GISAXS, AFM, Raman, and WCA data for various samples (PDF). Figures S1–S45.
