# Supplementary material for: Fabrication of Ultrafine, Highly Ordered Nanostructures Using Carbohydrate-Inorganic Hybrid Block Copolymers
Source: Nanomaterials (Basel). 2022 May 12;12(10):1653. doi: 10.3390/nano12101653 (PMC9144075; doi:10.3390/nano12101653)
Supplement: Supplementary file 1 [file nanomaterials-12-01653-s001.zip › nanomaterials-1714778-supplementary.pdf]

Supporting Information

Fabrication of Ultrafine, Highly Ordered Nanostructures Using Carbohydrate-Inorganic Hybrid Block Copolymers

Taiki Nishimura [1], Satoshi Katsuhara [1], Chaehun Lee [1], Brian J. Ree [2], Redouane Borsali [3], Takuya Yamamoto [2], Kenji Tajima [2], Toshifumi Satoh [2,*] and Takuya Isono [2,*]

[1] Graduate School of Chemical Sciences and Engineering, Hokkaido University, Sapporo 060-8628, Japan; taiki-nsm-1216@eis.hokudai.ac.jp (T.N.); satoshi-k@eis.hokudai.ac.jp (S.K.); dlcogns7942@eis.hokudai.ac.jp (C.L.)

[2] Division of Applied Chemistry, Faculty of Engineering, Hokkaido University, Sapporo 060-8628, Japan; brianree@eng.hokudai.ac.jp (B.J.R.); yamamoto.t@eng.hokudai.ac.jp (T.Y.); ktajima@eng.hokudai.ac.jp (K.T.)

[3] Centre de Recherches sur les Macromolécules Végétales (CERMAV), Centre National de la Recherche Scientifique (CNRS), Université Grenoble Alpes, F-38000 Grenoble, France; borsali@cermav.cnrs.fr

* Correspondence: satoh@eng.hokudai.ac.jp (T.S.); isono.t@eng.hokudai.ac.jp (T.I.)

Materials

6-Bromohexanoic Acid (>98.0%), 2-Propyne-1-ol (>98.0%), 3-Buten-1-ol (>98.0%), 1,1,3,3,5,5,7,7,9,9,11,11,13,13-Tetradecamethylheptasiloxane (DMS_n; >95.0%), 1-Ethyl-3-(3-dimethylaminopropyl)carbodiimide Hydrochloride (EDC; >98.0%), 4-dimethylaminopyridine (DMAP, >99.0%), *N,N,N',N'',N''*-Pentamethyldiethlenetriamine (PMDETA, >98.0%), Propargylamine (>97.0%), β-D-glucose pentaacetate ($AcGlc_1$; >97.0%), α-D-glucopyranosyl-β-D-glucose pentaacetate ($AcGlc_2$; >97.0%), Boron trifluoride-Diethyl Ether Complex ($BF_3 \cdot Et_2O$; >98.0%), and Sodium Methoxide (5 mol L^{-1} in methanol) were purchased from Tokyo Chemical Industry Co., Ltd., and used as received. Copper(I) Bromide (CuBr, 99.999%), Sodium Azide (NaN_3; >99.5%), Deuterium oxide (D_2O; >99.9%, contains 0.05 wt% 3-(trimethylsilyl)propionic-2,2,3,3-d_4 acid, soudium salt (DSS)) and Platinum(0)-1,3-divinyl-1,-1,3,3-tetramethyldisiloxane complex solution (Karstedt catalyst; Pt ~2% in xylene) were purchased from Sigma Aldrich and used as received. Sodium sulfate (Na_2SO_4, >99.0%), Celite®545, acetic anhydride (>97%), toluene (>99.0%), ethyl acetate (>99.3%), acetone (>99.0%), *n*-Hexane (>95.0%), Chloroform-*d* ($CDCl_3$; >99.8%), Dimethyl Sulfoxide-d_6 (DMSO-d_6; >99.9%), dry Tetrahydrofuran (dry THF;>99.5%, water content <0.001%), and dry *N,N*-dimethylformamide (dry DMF; >99.5%, water content <0.001%) were purchased from Kanto Chemical Co., Ltd., and used as received. Charcoal activated (powder), Dichloromethane (CH_2Cl_2; >98.0%), dry CH_2Cl_2 (>99.5%; water content, 99.0%), and *N,N*-dimethylformamide (DMF, >99.0%) were purchased from Junsei Chemical Co., Ltd., and used as received. Maltotriose was purchased from Hayashibara Co., Ltd., and

used as received. Dowex® 50WX2 hydrogen form was purchased from Sigma Aldrich and was washed with MeOH before use. 6-azidohexanoic acid was prepared according to previous reported methods.

Instruments

^{1}H NMR measurement

^{1}H NMR (400 MHz) spectra were obtained using a JEOL JNM-ECS 400 instrument at 25 °C.

Size exclusion chromatography (SEC)

SEC measurements were performed at 40 °C in DMF (flow rate, 0.6 mL min^{-1} ; containing 0.01 mol L^{-1} LiCl) using a Jasco HPLC system (PU-980 Intelligent HPLC Pump, CO-965 Column Oven, RI-930 Intelligent RI Detector, and DG-2080-53 Degasser) equipped with a Shodex KD-G guard column (4.6 mm × 10 mm; particle size, 8 μm), a Shodex Asahipak GF-310 HQ column (linear; particle size, 5 μm; 7.5 mm × 300 mm; exclusion limit, 4×10^{4}) and a Shodex Asahipak GF-7 M HQ column (linear; particle size, 9 μm; 7.5 mm × 300 mm; exclusion limit, 1.0×10^{7}). The number-average molecular weight ($M_{n,SEC}$) and dispersity ($Đ$) were calculated based on polymethyl methacylate standards.

Fourie transform infrared spectroscopy (FT-IR)

The FT-IR spectra were obtained using a PerkinElmer Frontier MIR spectrometer equipped with a S5 single reflection diamond universal attenuated total reflection (ATR) accessory.

Thermogravimetric analysis (TGA)

The TGA analysis was performed using Hitachi STA200RV under nitrogen atmosphere. All polymer samples were heated up to 600 °C at the heating rate of 10 °C min^{-1}.

Differential scanning calorimetry (DSC) measurement

The DSC experiments were performed using a Hitachi DSC 7000X under nitrogen atmosphere. All polymer samples were heated to 200 °C, cooled to −150 °C, and heated again to 200 °C at the heating and cooling rate of 10 °C min^{-1}.

Atomic force microscopy (AFM) measurement

The AFM phase images were obtained using a Molecular Imaging PicoPlus atomic force microscope operating in the tapping mode with a silicon cantilever (Nanoworld AG, NANOSENSORS™ PPP-NCH) having resonant frequency and spring constant of 190 kHz and 48 N m^{-1}, respectively. The thin films for the AFM observation were prepared by spin-coating (3,000 rpm for 60 s) the polymer solution in DMF (5% (w/w)) onto a Si substrate with a native oxide layer.

Small-angle X-ray scattering (SAXS)

The SAXS measurements of the obtained polymers were performed at the BL-6A beamline of the Photon Factory in the High Energy Accelerator Research Organization (KEK, Tsukuba, Japan) using X-ray beams with λ = 0.15 nm at room temperature. The scattering data were collected by a 2D detector (PILATUS3 1M (SAXS) (Dectris Ltd.)), where the samples-to-detector distance was set to be 1.5 m for SAXS measurement. The scattering angle (θ) was calibrated using silver behenate (Nagara Science Co., Ltd) as the standard and derived the scattering vector (q) from Bragg's equation ($|q|$ =

($4\pi/\lambda$) sin(θ/2)). The domain-spacing (d) value was calculated by $d = 2\pi/q^*$ (q^* is principal scattering peak position). The polymer films or powders were sandwiched by two pieces of Kapton tapes with a spacer of a stainless washer, which were applied for the measurement.

Wide-angle X-ray scattering (WAXS)

The WAXS measurements of the obtained polymers were performed at the BL-6A beamline of the Photon Factory in the High Energy Accelerator Research Organization (KEK, Tsukuba, Japan) using X-ray beams with λ = 0.15 nm at room temperature. The scattering data were collected by a 2D detector (PILATUS3 1M (WAXS) (Dectris Ltd.)), where the samples-to-detector distance was set to be 0 m for WAXS measurement. The scattering angle (θ) was calibrated using silver behenate (Nagara Science Co., Ltd) as the standard and derived the scattering vector (q) from Bragg's equation ($|q|$ = ($4\pi/\lambda$) sin(θ/2)). The domain-spacing (d) value was calculated by $d = 2\pi/q^*$ (q^* is principal scattering peak position). The polymer films or powders were sandwiched by two pieces of Kapton tapes with a spacer of a stainless washer, which were applied for the measurement.

Grazing incidence small-angle X-ray scattering (GISAXS)

GISAXS experiments for thin film samples were performed at BL-6A beamline of the Photon Factory in the High Energy Accelerator Research Organization (KEK, Tsukuba, Japan) using X-ray beams with λ = 0.15 nm at room temperature. A PILATUS3 1M (Dectris Ltd., Switzerland) detector, with 981 × 1043 pixels at a pixel size of 172 × 172 μm, and a counter depth of 20 bits (1,048,576 counts), was used for data acquisition. The sample-to-detector distance was calibrated using the

scattering patterns of silver behenate. The GISAXS profiles were acquired under ambient condition. At the PLA, scattering data were measured at room temperature in vacuum using X-ray radiation sources with a wavelength of 1.21 Å and a two-dimensional (2D) charge-coupled detector (CCD) (model Rayonix 2D SX 165, Rayonix, Evanston, IL, USA); each scattering image was normally collected for 10–30 s. The sample-to-detector distance was calibrated using the scattering patterns of silver behenate.

Raman microscope

Raman spectra were measured by a Raman spectrometer (XploRA PLUS, Horiba Scientific) to identify structural unites of the thin films samples on the silicon wafer. The excitation and intensity of the laser beam were 532 nm and 17 mW, respectively.

Water Contact Angle measurement

Static water contact angle measurements were performed on a DropMaster (DMe-201, Kyowa Interface Science Co. Ltd., Saitama, Japan) in an ambient environment of room temperature. The needle diameter was 0.47 mm, and the water droplet volume was set to ~5 µL. Each measurement was repeated 5 times for a better statistical significance.

Synthesis

Synthesis of α,ω-dihydroxyl-functionalized *o*DMS (HO–DMS$_n$–OH)

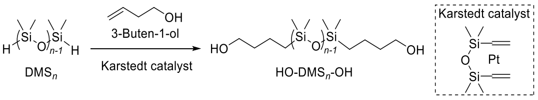

DMS$_n$ (1.00 g, 1.98 mmol) and 3-buten-1-ol (1.07g, 14.9 mmol) were dissolved in dry-toluene (180 mL) and added to a two-necked flask. The mixture was stirred under an argon atmosphere at ambient temperature. After 30 min, Karstedt catalyst (0.30 mL, 0.95 mmol as a ~2% stock solution in xylene) was added and the mixture was stirred for additional 4 h at ambient temperature. Then activated carbon was added and stirred for 18 h and catalyst was removed with celite filtration. The obtain product was evaporated to remove the excessive 3-buten-1-ol and dry-toluene. Finally, HO–DMS$_n$–OH was obtained as a colorless oil (2.66 g). Yield: 68.9 %

^{1}H NMR (400 MHz, $CDCl_3$): δ (ppm) 3.67 (t, J = 6.4 Hz, 4H, –$C\underline{H}_2OH$), 1.67–1.58 (m, 4H, –$C\underline{H}_2CH_2OH$), 1.49–1.40 (m, 4H, –$C\underline{H}_2CH_2CH_2OH$), 0.62-0.57 (m, 4H, –$C\underline{H}_2CH_2CH_2CH_2OH$), 0.27–(−0.02) (m, 42H, –$C\underline{H}_3$).

^{13}C NMR (100 MHz, $CDCl_3$): δ (ppm) 62.9 (–$\underline{C}H_2OH$), 36.5 (–$\underline{C}H_2CH_2OH$), 19.6 (–$\underline{C}H_2CH_2CH_2CH_2OH$), 18.1 (–$\underline{C}H_2CH_2CH_2OH$), 1.3 (–$CH_2Si(\underline{C}H_3)_2$–) 1.2 (–$OSi(\underline{C}H_3)_2O$–).

HRMS (ESI): Calcd. for $C_{22}H_{60}O_8Si_7Na^+$ $[M+Na]^+$: 671.25651, found: 671.25653.

Synthesis of α,ω-diazido-functionalized *o*DMS (N_3–DMS$_n$–N_3)

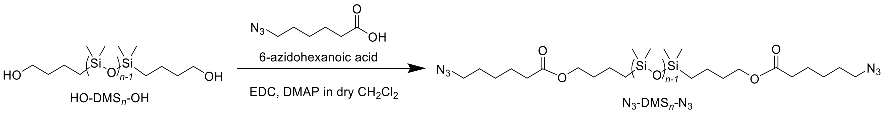

EDC (4.46 g, 23.3 mmol), DMAP (2.85 g, 23.3 mmol), HO–DMS$_n$–OH (2.50 g, 3.85 mmol), and 6-azidohexanoic acid (3.59 g, 22.8 mmol) were dissolved in dry-CH_2Cl_2 (30 mL) and added to a round -bottom flask. The mixture was stirred at ambient temperature. After 48 h, the mixture was evaporated to remove dry-CH_2Cl_2. The obtain product was dissolved in *n*-hexane and washed with methanol for three times. The residue was evaporated to remove *n*-hexane. Then, N_3–DMS$_n$–N_3 was obtained as a colorless oil (2.37 g). Yield: 66.4 %

^{1}H NMR (400 MHz, $CDCl_3$): δ (ppm) 4.09 (t, J = 6.9 Hz, 4H, –C$\underline{H}_2$O–), 3.30 (t, J = 6.9 Hz, 4H, –C$\underline{H}_2N_3$), 2.34 (t, J = 7.3 Hz, 4H, –C(=O)C$\underline{H}_2$–), 1.75–1.58 (m, 12H, –C$\underline{H}_2$CH$_2$C$\underline{H}_2$C$\underline{H}_2$CH$_2N_3$), 1.48–1.35 (m, 8H, –C$\underline{H}_2$C$\underline{H}_2$CH$_2$O–), 0.62–0.55 (m, 4H, –Si(CH$_3$)$_2$C$\underline{H}_2$–), 0.13–0.05 (m, 42H, –C$\underline{H}_3$).

^{13}C NMR (100 MHz, $CDCl_3$): δ (ppm) 173.7 (–$\underline{C}$=O–), 64.3 (–$\underline{C}$H$_2$O–), 51.4 (–$\underline{C}$H$_2N_3$), 34.2 (–$\underline{C}$H$_2$CH$_2N_3$), 32.2 (–$\underline{C}$H$_2$CH$_2$O–), 28.7 (–Si(CH$_3$)$_2\underline{C}$H$_2$–), 26.4 (–C(=O)$\underline{C}$H$_2$–), 24.6 (–$\underline{C}$H$_2$CH$_2$CH$_2N_3$), 19.8 (–$\underline{C}$H$_2$CH$_2$CH$_2$O–), 17.9 (–C(=O)CH$_2\underline{C}$H$_2$–), 1.3 (–CH$_2$Si($\underline{C}$H$_3$)$_2$–) 1.2 (–OSi($\underline{C}$H$_3$)$_2$O–).

HRMS (ESI): Calcd. for $C_{34}H_{78}N_6O_{10}Si_7\ Na^+$ $[M+Na]^+$: 949.40524, found: 949.40565.

Synthesis of 2-propyne-1-yl-β-D-glucopyranoside (Glc_1–C≡CH)

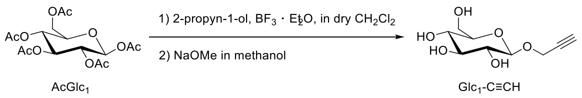

A typical procedure for the synthesis of $AcGlc_m$–C≡CH (m = 1, 2) is as follows (method A). *β*-D-glucose pentaacetate ($AcGlc_1$) (20.0 g, 51.2 mmol) and propargyl alcohol (3.63 mL, 61.4 mmol) were dissolved in dichloromethane under nitrogen atmosphere. After cooling down to 0 °C, boron trifluoride-ethyl ether complex (9.65 mL, 76.8 mmol) was added to the solution, and the whole mixture was stirred at room temperature for 2 h. Potassium carbonate was added to the reaction mixture, which was stirred for additional 30 min. The mixture was filtered and washed by water to give 2-propynyl-*β*-D-glucose tetraacetate ($AcGlc_1$–C≡CH).

The deacetylation of $AcGlc_1$–C≡CH was conducted as follows (method B). Sodium methoxide (5 mol L^{-1} in methanol, 100 μL, 500 μmol) was added to a solution of $AcGlc_1$–C≡CH (10.0 g, 26 mmol) in methanol (50 mL), and the whole mixture was stirred at 0 °C for 1 h. After stirring at room temperature for additional 24 h, Dowex®50WX2 hydrogen form was added to adjust the pH to be 6−7. After removing Dowex® by filtration, the solvent was evaporated under reduced pressure. The residue was purified by recrystallization from methanol to give 2-propyne-1-yl-*β*-D-glucopyranoside (Glc_1–C≡CH) as a white solid. Yield: 79 %

^{1}H NMR (400 MHz, DMSO-d_6): δ (ppm) 5.14 (d, J = 4.9 Hz, 1H, H-1), 4.99 (d, J = 4.9 Hz, 1H, H-4), 4.94 (d, J = 5.2 Hz, 1H, H-5), 4.53 (t, J = 6.1 Hz, 1H, H-2), 4.38 (dd, J = 15.5, 2.5 Hz, 1H, H-3), 4.26-4.23 (m, 2H, H-6), 4.11 (q, J = 5.2 Hz, 2H, –$C\underline{H}_2$C≡CH), 3.71–3.61 (m, J = 11.8, 6.6, 1.9 Hz, 1H, –

C≡C<u>H</u>).

Synthesis of 2-propyne-1-yl-4-*O*-α-D-glucopyranosyl-β-D-glucopyranodo (Glc_2–C≡CH)

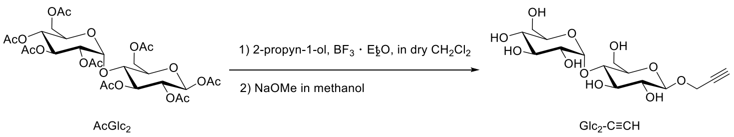

Method A was used for the addition reaction of *α*-D-glucopyranosyl-*β*-D-glucose pentaacetate ($AcGlc_2$) (30.0 g, 44.2 mmol) and propargyl alcohol (3.00 g, 53.6 mmol) in dichloromethane (200 mL) with boron trifluoride-ethyl ether complex (10 mL, 56 mmol) as a catalyst under nitrogen atmosphere. The whole mixture was stirred at room temperature for 2 h, and potassium carbonate was added to the reaction mixture, which was stirred for additional 30 min. The resulting product was purified by filtration and washed by water. Then Method B was used for the deacetylation of 2-propyne-1-yl-4-*O*-*α*-D-glucopyranosyl-*β*-D-glucose tetraacetate ($AcGlc_2$–C≡CH; 15.6 g, 23.0 mmol) in methanol (250 mL) with sodium methoxide (5 mol L^{-1} in methanol, 920 μL, 4.60 mmol) as a catalyst, and the whole mixture was stirred at 0 °C for 1 h. After stirring at room temperature for additional 24 h, Dowex®50WX2 hydrogen form was added to adjust the pH to be 6−7. The residue was purified by recrystallization from methanol to give 2-propyne-1-yl-4-*O*-*α*-D-glucopyranosyl-*β*-D-glucopyranoside (Glc_2–C≡CH) as a white solid. Yield: 52.2 %

^{1}H NMR (400 MHz, DMSO-d_6): *δ* (ppm) 5.36–5.19 (m, 4H, H-1, -4$^{GlcI,\ II}$), 4.98 (t, *J* = 9.8 Hz 1H, H-5^{GlcI}), 4.89–4.84 (m, 2H, H-2^{GlcI}, -5^{GlcII}) 4.67 (dd, *J* = 9.4, 8.0 Hz, 1H, H-2^{GlcI}), 4.40–4.12 (m, 6H, H-3, -6$^{GlcI,\ II}$), 4.06–3.90 (m, 2H, –C<u>H</u>$_2$C≡CH), 3.56–3.51 (m, 1H, –CH$_2$C≡C<u>H</u>), 2.08–1.94 (m, 21H, –C<u>H</u>$_3$).

Synthesis of *N*-maltotriose-3-acetamido-1-propyne (Glc_3–C≡CH)

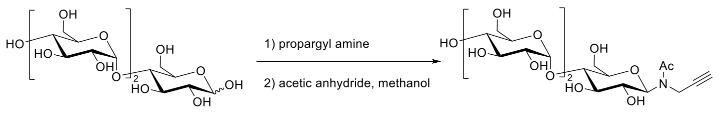

Glc_3 Glc_3-C≡CH

The end functional of a maltotriose was conducted according to previously established method. In this reaction, propargylamine reacts only with the hemiacetal reducing end of the maltotriose, leading to the intermediate is then treated with acetic anhydride to produce stable Glc_3–C≡CH. Maltotriose (1.52 g, 3.01 mmol) and propargylamine (4.24 mL, 66.3 mmol) were added to a round-bottom flask and the mixture was stirred under an argon atmosphere at ambient temperature. After 72 h, the reaction mixture was precipitated into CH_2Cl_2. The precipitate was filtered and washed with CH_2Cl_2. The obtained precipitate was dissolved in a mixture of acetic anhydride and methanol (1/5 (v/v), 240 mL) and stirred at ambient temperature. After 48 h, the excess of acetic anhydride was removed by co-evaporation with a mixed solvent of toluene and methanol. The resulting residue was precipitated into CH_2Cl_2 to give Glc_3–C≡CH as a white solid (1.47 g). Yield 90.1 %

^{1}H NMR (400 MHz, D_2O): δ (ppm) 5.54 and 5.09 (rotamers, 1H, –CHNAc), 5.39–5.44 (m, 2H, H-1), 4.32–3.24 (m, 20H, H-2, -3, -4, -5, -6, –NCH_2), 2.74 and 2.58 (t, 1H, rotamers, J = 2.3 Hz, –C≡CH), 2.32 and 2.25 (s, rotamer, 3H, –CH_3).

^{13}C NMR (100 MHz, D_2O): δ (ppm) 177.2 (–$COCH_3$), 102.4 and 102.1 (C-$1^{\mathrm{GlcII,\ III}}$), 89.0 (C-1^{GlcI}), 79.7, 79.4, 79.3, 79.1, 78.5, 75.9, 75.5, 75.3, 74.3, 74.1, 73.8, 72.8, and 71.9 (C-2, -3, -4, -$5^{\mathrm{GlcI,\ II,\ III}}$, C-$6^{\mathrm{GlcI}}$, –C≡CH, –C≡CH), 63.2 and 63.0 (C-$6^{\mathrm{GlcII,\ III}}$), 41.7 (–NCH_2), 23.8 (–CH_3).

LRMS (ESI): Calcd. for $C_{23}H_{37}NO_{16}Na^+$ $[M+Na]^+$ 606.20, found 606.20.

Synthesis of Glc$_1$-*b*-DMS$_n$-*b*-Glc$_1$

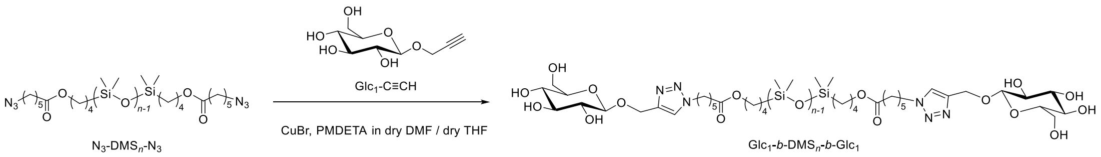

A typical click reaction procedure is as follows (method C). Glc$_1$–C≡CH (497 mg, 2.28 mmol) and copper(I) bromide (CuBr; 114 mg, 0.796 mmol) were placed into Schlenck flask, which was evacuated and back-filled with argon three times. A solution of N_3–DMS$_n$–N_3 (700 mg, 0.755 mol) and *N*,*N*,*N'*,*N''*,*N''*-pentamethyldiethylenetriamine (PMDETA; 180 μL, 0.75 mol) in a mixed solvent of dry DMF (15 mL) and dry THF (15 mL) was degassed by the argon bubbling, and the mixture was stirred at 60 °C for 24 h and treated with Dowex®50WX2 hydrogen form to remove Cu catalyst. The resulting product was purified by Reversed-phase silica gel chromatography (H_2O, 100mL; H_2O/methanol = 9/1 (v/v), 100 mL; H_2O/methanol = 3/7 (v/v), 100 mL; H_2O/methanol = 1/1 (v/v), 100mL; H_2O/methanol = 1/4 (v/v), 100 mL; methanol, 100 mL; methanol/acetone = 1/1 (v/v), 100 mL; acetone, 100 mL) and the solvent was evaporated under reduced pressure. The residue was dissolved into DMF and removed the others impurities by filtration with membrane filter (pore diameter is 0.45 μm). Then, Glc$_1$-*b*-DMS$_n$-*b*-Glc$_1$ was obtained as a yellow solid (0.253 g). Yield: 24.3 %

^{1}H NMR (400 MHz, DMSO-d_6): δ (ppm) 8.10 (s, 2H, –NC<u>H</u>=C(N–)–), 5.02 (d, J = 5.0 Hz, 2H,H-1), 4.93 (q, J = 5.0 Hz, 2H,H-4), 4.83 (d, J = 12.3 Hz, 2H,H-5), 4.67–4.47 (m, 4H,H-6), 4.31 (t, J = 7.1 Hz, 4H, –C<u>H</u>$_2$OGlc$_1$), 4.24 (d, J = 7.8 Hz, 2H, H-2),3.98 (t, J = 6.4 Hz, 4H, –C<u>H</u>$_2$OC(=O)–) 3.70 (q, J = 5.5 Hz, 2H, H-3), 3.19–3.09 (m, 4H, –CH$_2$C<u>H</u>$_2$N–), 3.08–2.94 (m, 4H,–OH-2, -3, -4, -6), 2.34–2.18

(m, 4H, –C(=O)CH_2–), 1.86–1.71 (quin, J=7.3 Hz, 4H, –CH_2CH_2N–), 1.65–1.46 (m, 8H, –CH_2CH_2O–, –C(=O)CH_2CH_2–), 1.40–1.13 (m, 8H, –$CH_2CH_2CH_2$O–, –$CH_2CH_2CH_2$N–), 0.53 (t, J = 8.2 Hz, 4H, –Si$(CH_3)_2CH_2$–), 0.07–(−0.12) (m, 62H, –CH_3).

^{13}C NMR (100 MHz, DMSO-d_6): δ (ppm) 172.7 (–C(=O)–), 143.7 (–NCH=C(N–)CH_2–), 124.1 (–NCH=C(N–)CH_2–), 102.2 (C-1), 77.0 (C-2), 76.7 (C-3), 73.4 (C-5), 70.1 (–CH_2OGlc$_1$), 63.3 (–CH_2CH_2O–), 61.6 (C-6), 61.2 (C-4), 49.1 (–CH_2N–), 33.3 (–C(=O)CH_2–), 31.6 (–CH_2CH_2O–), 29.4 (–CH_2CH_2N–), 25.4 (–$CH_2CH_2CH_2$N–), 23.9 (–CH_2CH_2C(=O)–), 19.2 (–Si$(CH_3)_2CH_2$–), 17.2 (–$CH_2CH_2CH_2$O–), 1.0 (–CH_2Si$(CH_3)_2$–), 0.1(–OSi$(CH_3)_2$O–).

Synthesis of Glc$_2$-*b*-DMS$_n$-*b*-Glc$_2$

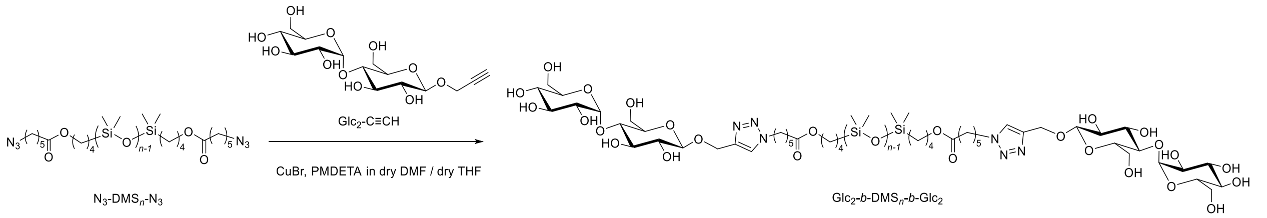

Method C was used for the click reaction of N_3–DMS$_n$–N_3 (700 mg, 0.755 mol) and Glc$_2$–C≡CH (867 mg, 2.28 mmol) in a mixed solvent of DMF (15 mL) and THF (15 mL) with CuBr (114 g, 0.796 mol) and PMDETA (180 µL, 0.75 mol) as a catalyst and a regard, respectively. The resulting product was purified by Reversed-phase silica gel chromatography (H_2O, 100mL; H_2O/methanol = 9/1 (v/v), 100 mL; H_2O/methanol = 3/7 (v/v), 100 mL; H_2O/methanol = 1/1 (v/v), 100mL; H_2O/methanol = 1/4 (v/v), 100 mL; methanol, 100 mL; methanol/acetone = 1/1 (v/v), 100 mL; acetone, 100 mL) and the solvent was evaporated under reduced pressure. The residue was dissolved into DMF and removed

the others impurities by filtration with membrane filter (pore diameter is 0.45 μm). Then, Glc$_2$-*b*-DMS$_n$-*b*-Glc$_2$ was obtained as a yellow powder (0.298 g). Yield: 23.6 %

^{1}H NMR (400 MHz, DMSO-d_6): δ (ppm) 8.12–8.09 (br, 2H, –NC$\underline{\mathrm{H}}$=C(N–)–), 5.50 (d, J = 3.2 Hz, 2H, H-1$^{\mathrm{GlcI,}}$), 5.43 (d, J = 6.4 Hz, 2H, H-1^{GlcII}), 5.14 (d, J = 5.0 Hz, 2H, H-4^{GlcI}), 5.02 (d, J = 3.7 Hz, 2H, H-4^{GlcII}), 4.94–4.80 (m, 8H, H-2, -5$^{\mathrm{GlcI,\ II}}$), 4.63–4.46 (m, 8H, H-6$^{\mathrm{GlcI,\ II}}$), 4.31 (t, J = 7.5 Hz, 4H, –C$\underline{\mathrm{H}}_2$OGlc$_2$), 3.99 (t, J = 6.6 Hz, 4H, – C$\underline{\mathrm{H}}_2$OC(=O)–), 3.75 (q, J = 5.6 Hz 2H, H-3^{GlcI}), 3.67–3.54 (m, 2H, H-3$^{\mathrm{GlcII,}}$) 3.51–3.19 (m, 14H, –OH-2,3,4,6), 3.11–3.00 (m, 4H, –CH$_2$C$\underline{\mathrm{H}}_2$N–), 2.26 (t, J = 7.3 Hz, 4H, –C(=O)C$\underline{\mathrm{H}}_2$–), 1.80 (quin, J = 7.5 Hz 4H, –C$\underline{\mathrm{H}}_2$CH$_2$N–), 1.60–1.50 (m, 8H, –C$\underline{\mathrm{H}}_2$CH$_2$O–, –C(=O)CH$_2$C$\underline{\mathrm{H}}_2$–), 1.40–1.18 (m, 8H, –C$\underline{\mathrm{H}}_2$CH$_2$CH$_2$O–, –C$\underline{\mathrm{H}}_2$CH$_2$CH$_2$N–), 0.53 (t, J = 8.2 Hz, 4H, –Si(CH$_3$)$_2$C$\underline{\mathrm{H}}_2$–), 0.12–(−0.04) (m, 63H, –C$\underline{\mathrm{H}}_3$).

^{13}C NMR (100 MHz, DMSO-d_6): δ (ppm) 172.7 (–$\underline{\mathrm{C}}$=O–), 143.5 (–NCH=$\underline{\mathrm{C}}$(–N)CH$_2$–), 124.0 (–N$\underline{\mathrm{C}}$H=C(N–)CH$_2$–), 101.9 and 100.7 (C-1), 79.6 and 76.4 (C-2), 75.2 and 73.4 (C-3), 72.9 and 72.4 (C-5), 69.6 (–$\underline{\mathrm{C}}$H$_2$OGlc$_2$), 63.3 (–CH$_2\underline{\mathrm{C}}$H$_2$O–), 61.7 (C-6), 60.8 and 60.7 (C-4), 49.2 (–CH$_2\underline{\mathrm{C}}$H$_2$N–), 33.2 (–C(=O)$\underline{\mathrm{C}}$H$_2$–), 31.8 (–$\underline{\mathrm{C}}$H$_2$CH$_2$O–), 29.4 (–$\underline{\mathrm{C}}$H$_2$CH$_2$N–), 25.4 (–$\underline{\mathrm{C}}$H$_2$CH$_2$CH$_2$N–), 23.9 (–$\underline{\mathrm{C}}$H$_2$CH$_2$C(=O)–), 19.1 (–Si(CH$_3$)$_2\underline{\mathrm{C}}$H$_2$–), 17.2 (–$\underline{\mathrm{C}}$H$_2$CH$_2$CH$_2$O–), 1.0 (–CH$_2$Si($\underline{\mathrm{C}}$H$_3$)$_2$–), 0.1(–OSi($\underline{\mathrm{C}}$H$_3$)$_2$O–).

Synthesis of Glc_3-*b*-DMS_n-*b*-Glc_3

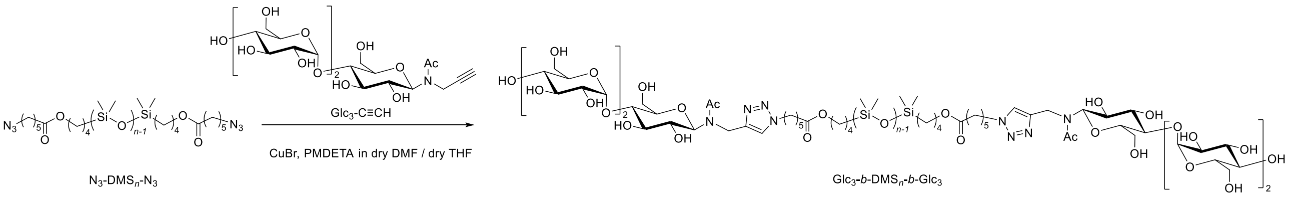

Method C was used for the click reaction of N_3–DMS_n–N_3 (700 mg, 0.755 mmol) and Glc_3–C≡CH (1.32 g, 2.26 mol) in a mixed solvent of DMF (15 mL) and THF (15 mL) with CuBr (114 mg, 0.796 mol) and PMDETA (180 μL, 0.75 mol) as a catalyst and a regard, respectively. The resulting product was purified by Reversed-phase silica gel chromatography (H_2O, 100mL; H_2O/methanol = 9/1 (v/v), 100 mL; H_2O/methanol = 3/7 (v/v), 100 mL; H_2O/methanol = 1/1 (v/v), 100mL; H_2O/methanol = 1/4 (v/v), 100 mL; methanol, 100 mL; methanol/acetone = 1/1 (v/v), 100 mL; acetone, 100 mL) and the solvent was evaporated under reduced pressure. The residue was dissolved into DMF and removed the others impurities by filtration with membrane filter (pore diameter is 0.45 μm). Then, Glc_3-*b*-DMS_n-*b*-Glc_3 was obtained as a brown powder (0.542 g). Yield: 40.3 %

^{1}H NMR (400 MHz, DMSO-d_6): δ (ppm) 8.10 and 8.01 (1H, –NCH=C(N–)–), 7.89 (s, 1H, –NCH=C(N–)–), 5.74–5.62 (m, 4H, H-$1^{GlcI, II}$), 5.59 (d, J = 6.4 Hz, 2H, H-1^{GlcIII}) 5.52–5.39 (m, 6H, H-$4^{GlcI, II, III}$), 5.01 (dd, J = 11.4, 3.2 Hz, 6H, H-$5^{GlcI, II, III}$), 4.95–4.80 (m, 6H, H-$2^{GlcI, II, III}$), 4.63–4.41 (m, 12H, H-$6^{GlcI, II, III}$), 4.32–4.22 (m, 4H, –CH_2NC(=O)CH_3), 3.99 (t, J = 6.4 Hz, 4H, –CH_2O(C=O)–), 3.76–3.18 (m, 28H, H-$3^{GlcI, II, III}$, –OH-2, -3, -4, -6), 3.06 (td, J = 9.1, 5.9 Hz, 4H, –CH_2CH_2N–), 2.25 (t, J = 7.5 Hz, 4H, –C(=O)CH_2–), 2.09 (s, 6H, –NC(=O)–CH_3), 1.78 (quin, J = 7.3 Hz, 4H, –CH_2CH_2N-–), 1.61–1.49 (m, 8H, –CH_2CH_2O–, C(=O)CH_2CH_2–), 1.40–1.14 (m, 8H, –$CH_2CH_2CH_2$O–, –

$C\underline{H}_2CH_2CH_2N$–), 0.53 (t, J = 8.5 Hz, 4H, – $Si(CH_3)_2C\underline{H}_2$–), 0.14–(−0.05) (m, 63H, $–SiC\underline{H}_3$).

^{13}C NMR (100 MHz, DMSO-d_6): δ (ppm) 172.7 ($–CH_2\underline{C}=O$–), 170.7 ($–N\underline{C}(=O)$–), 145.1 (–NCH=$\underline{C}$(N–)CH_2–), 123.4 (–N$\underline{C}$H=C(N–)CH_2–), 100.8 and 100.7 (C-1), 86.8, 79.6, and 79.5 (C-2), 77.3 and 76.3 (C-3), 73.5, 72.5, and 72.0 (C-5), 70.0 ($–\underline{C}H_2NAcGlc_3$), 63.3 ($–CH_2\underline{C}H_2O$–), 60.8 (C-6), 60.5 and 60.3 (C-4), 49.2 ($–CH_2\underline{C}H_2N$–), 33.2 ($–C(=O)\underline{C}H_2$–), 31.6 ($–\underline{C}H_2CH_2O$–), 29.4 ($–\underline{C}H_2CH_2N$–), 25.4 ($–\underline{C}H_2CH_2CH_2N$–), 23.9 ($–\underline{C}(=O)CH_3$), 21.8 ($–\underline{C}H_2CH_2C(=O)$–), 19.2 ($–Si(CH_3)_2\underline{C}H_2$–), 17.2 ($–\underline{C}H_2CH_2CH_2O$–), 1.1 ($–Si\underline{C}H_3$).

Etching condition

Etching experiments were performed by three process, first process was conducted with O_2 plasma 15 sccm, 10 mT, 250 W, 30 Wb, 15 s, second process was conducted with Ar plasma 80 sccm, O_2 plasma 40 sccm, 10 mT, 200 W, 20 Wb, 35 s, and final process was conducted with CF_4 plasma 60 sccm, O_2 plasma 60 sccm, 10 mT, 250 W, 30 Wb, 35 s.

All etching experiments were performed by RIE (Reactive Ion Etching) Plasma Etching System (RIE-10NR, Samco International, Kyoto, Japan) in the Open Fcility Center, Creative Research Institution, Hokkaido University.

Result

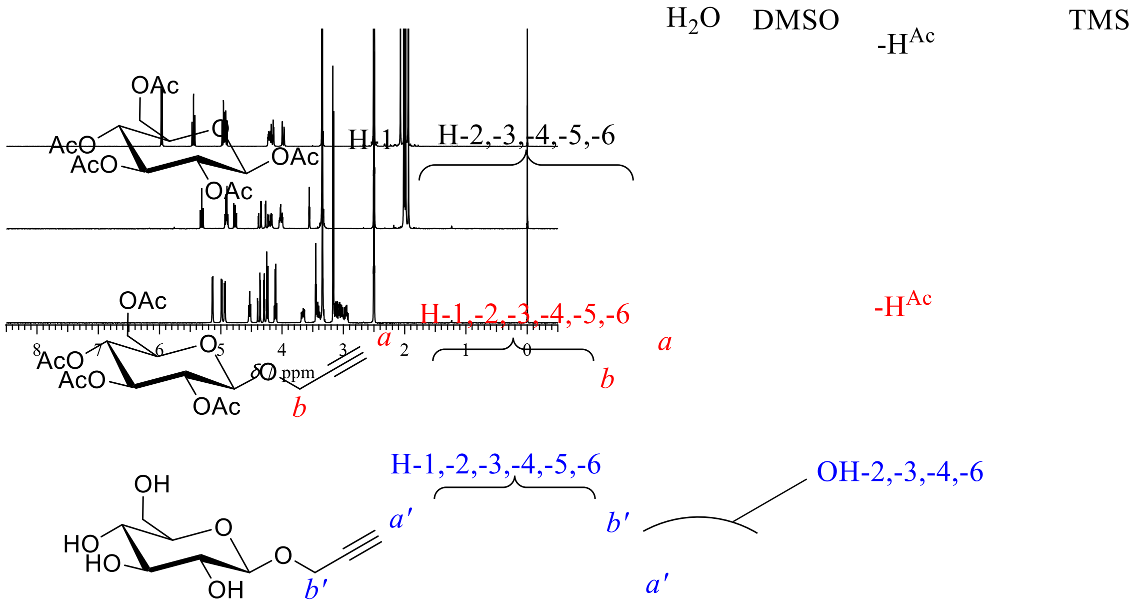

Figure S1. ^{1}H NMR spectra of AcGlc1 (upper), AcGlc1–C≡CH (middle), and Glc1–C≡CH (lower) in DMSO-*d*6 (400 MHz).

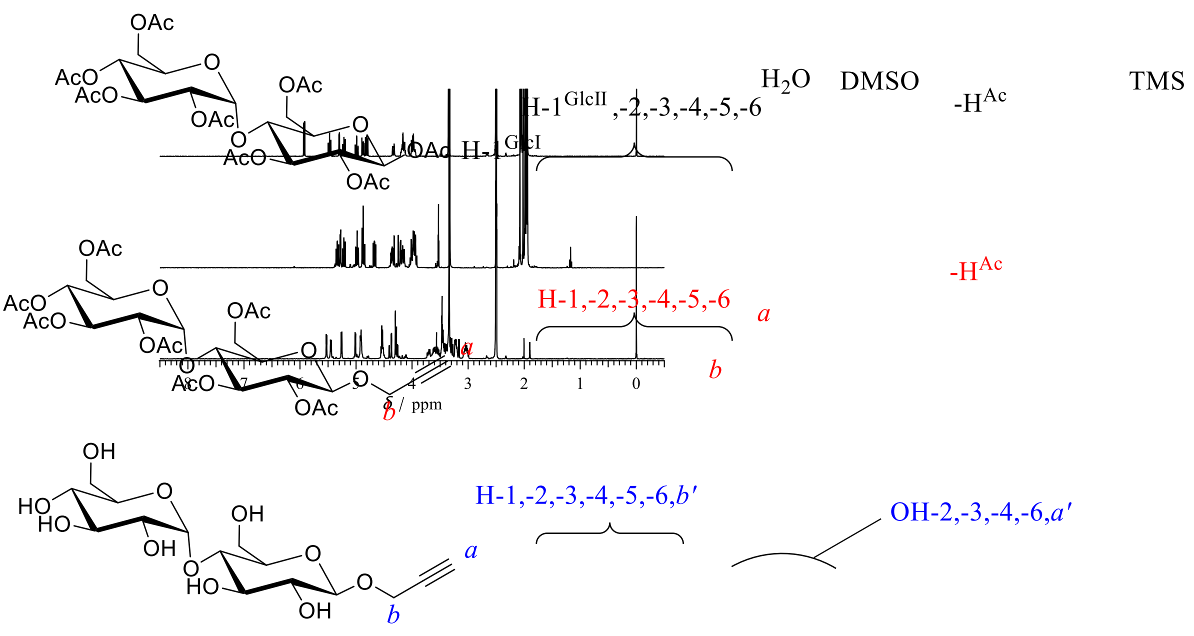

Figure S2. ^{1}H NMR spectra of AcGlc2 (upper), AcGlc2–C≡CH (middle), and Glc2–C≡CH (lower) in DMSO-*d*6 (400 MHz).

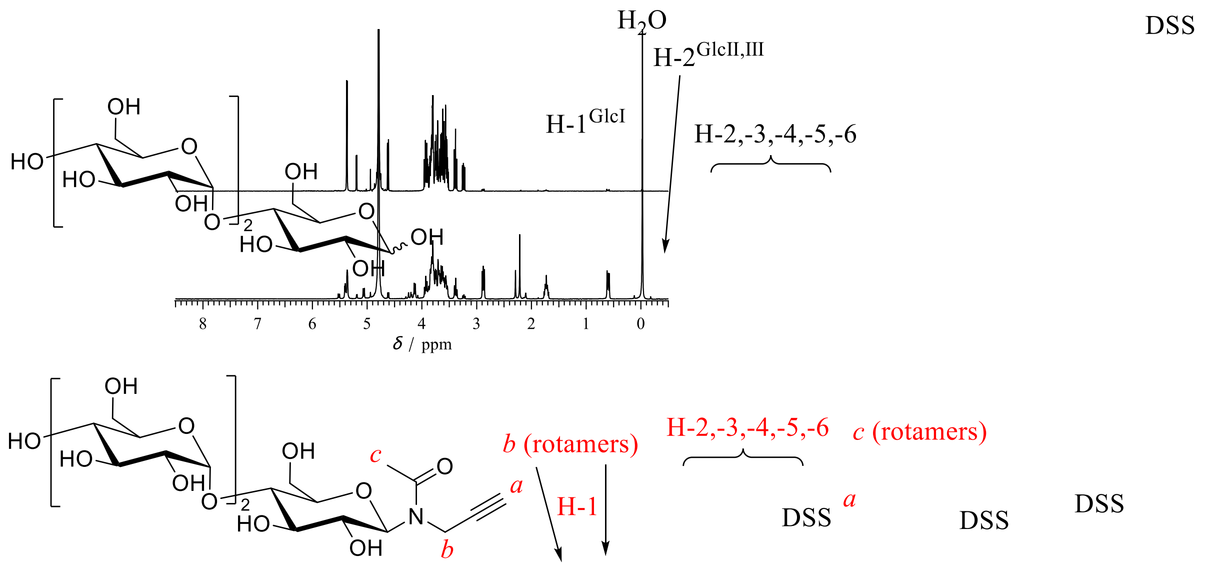

Figure S3. ^{1}H NMR spectra of Glc$_3$ (upper) and Glc$_3$–C≡CH (lower) in D_2O (400 MHz).

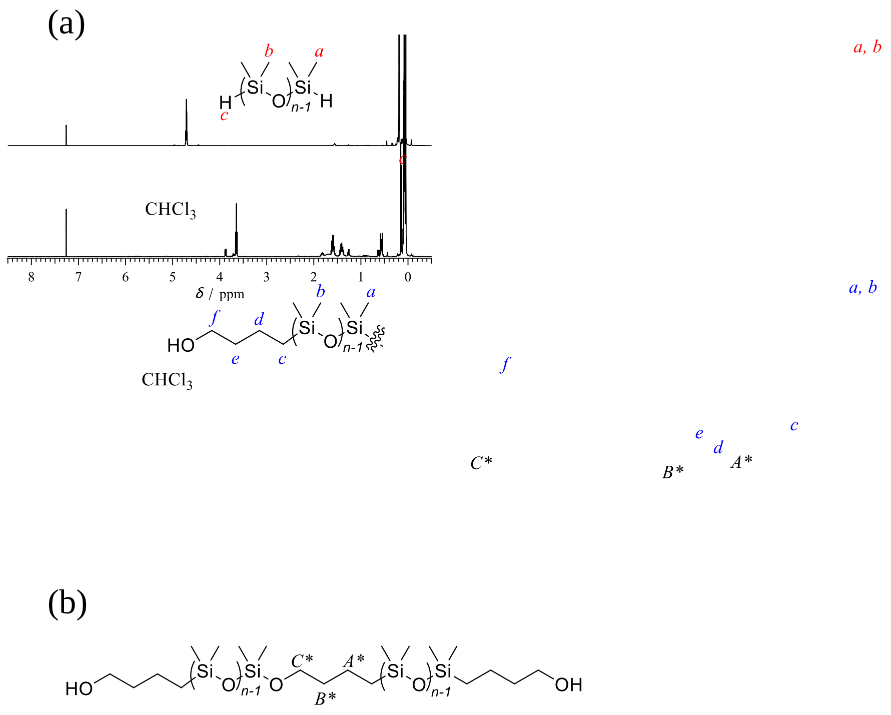

Figure S4. (**a**) ^{1}H NMR spectra of DMS$_n$ (upper) and HO–DMS$_n$–OH (lower) in $CDCl_3$ (400 MHz).

(**b**) The expected structure of the byproduct.

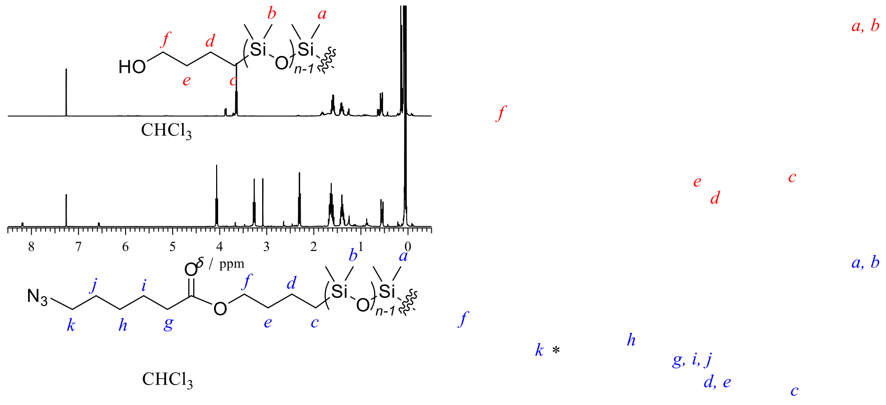

Figure S5. ^{1}H NMR spectra of HO–DMS$_n$–OH (upper) and N_3–DMS$_n$–N_3 (lower) in $CDCl_3$ (400 MHz).

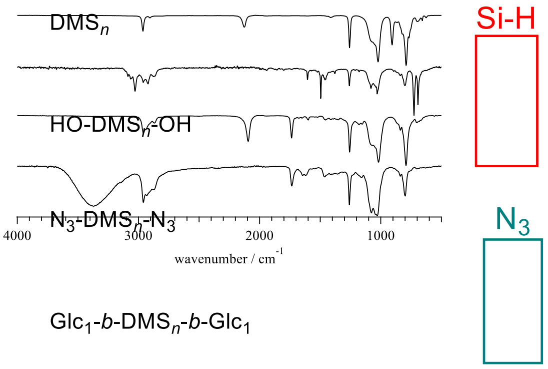

Figure S6. FT-IR spectra of DMS$_n$, HO–DMS$_n$–OH, N_3–DMS$_n$–N_3, and Glc$_1$-*b*-DMS$_n$-*b*-Glc$_1$.

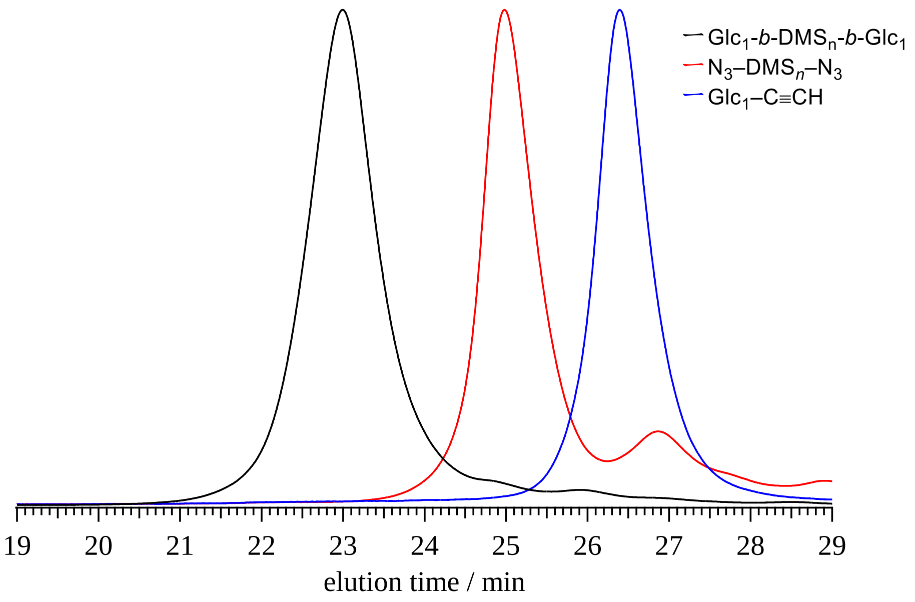

Figure S7. SEC traces of N_3–DMS$_n$–N_3 (red), Glc$_2$–C≡CH (blue), and Glc$_1$-*b*-DMS$_n$-*b*-Glc$_1$ (black).

(eluent, DMF containing 0.01 mol L^{-1} LiCl; flow rate, 0.60 mL min^{-1}, PMMA std.).

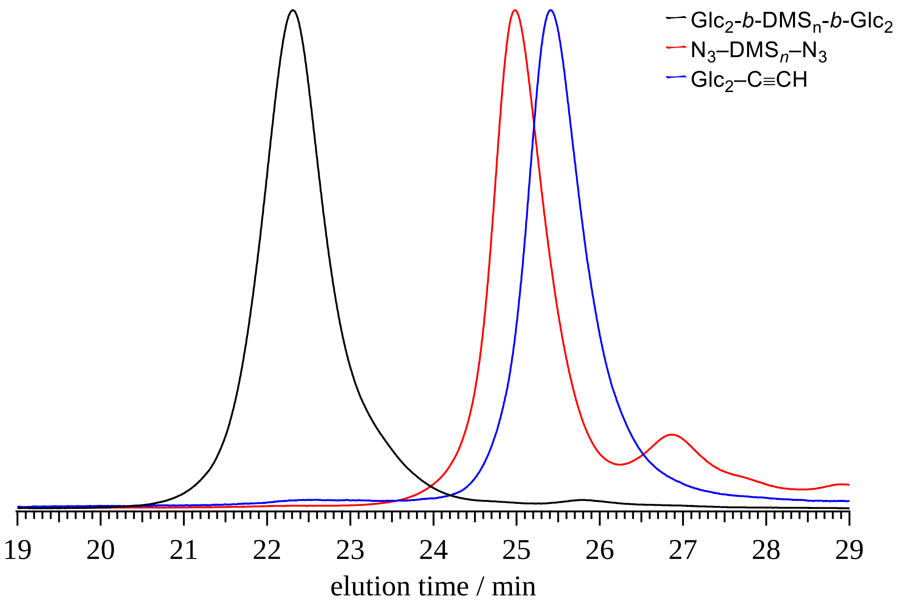

Figure S8. SEC traces of N_3–DMS$_n$–N_3 (red), Glc$_2$–C≡CH (blue), and Glc$_2$-*b*-DMS$_n$-*b*-Glc$_2$ (black).

(eluent, DMF containing 0.01 mol L^{-1} LiCl; flow rate, 0.60 mL min^{-1}, PMMA std.).

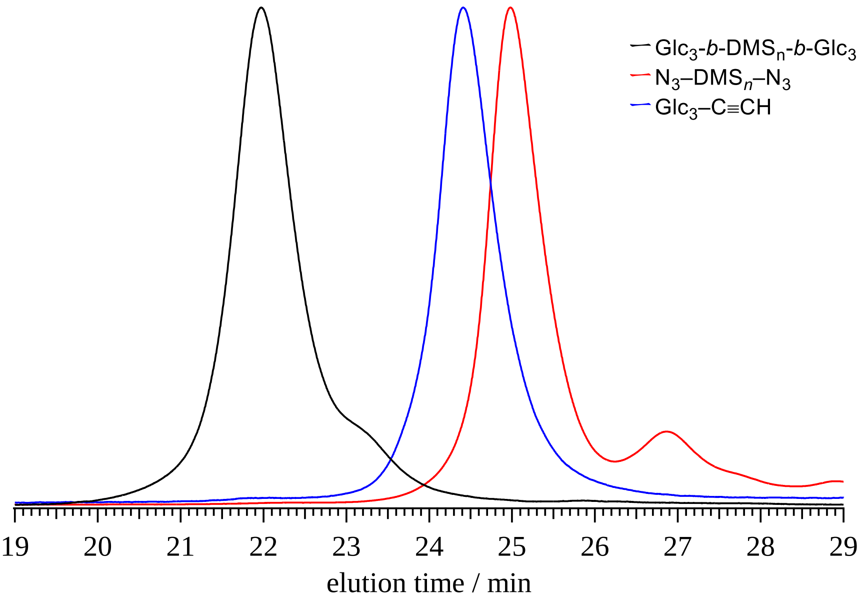

Figure S9. SEC traces of N_3–DMS$_n$–N_3 (red), Glc_3–C≡CH (blue), and Glc_3-*b*-DMS$_n$-*b*-Glc_3 (black). (eluent, DMF containing 0.01 mol L^{-1} LiCl; flow rate, 0.60 mL min^{-1}, PMMA std.).

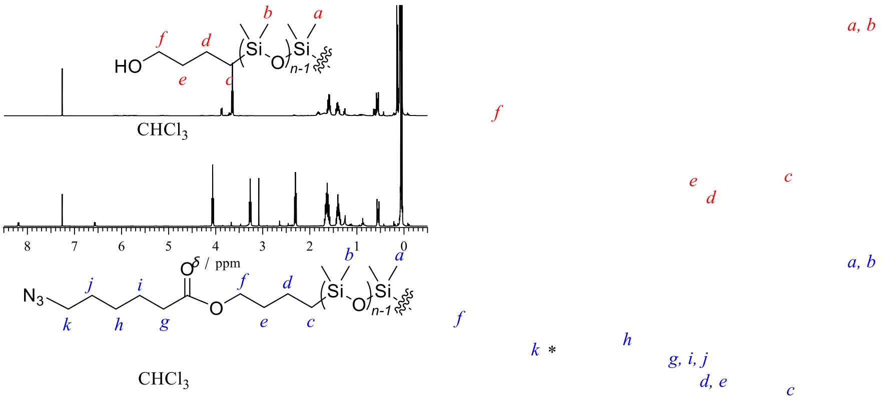

Figure S10. ^1H NMR spectra of HO–DMS$_n$–OH (upper) and N_3–DMS$_n$–N_3 (lower) in $CDCl_3$ (400 MHz).

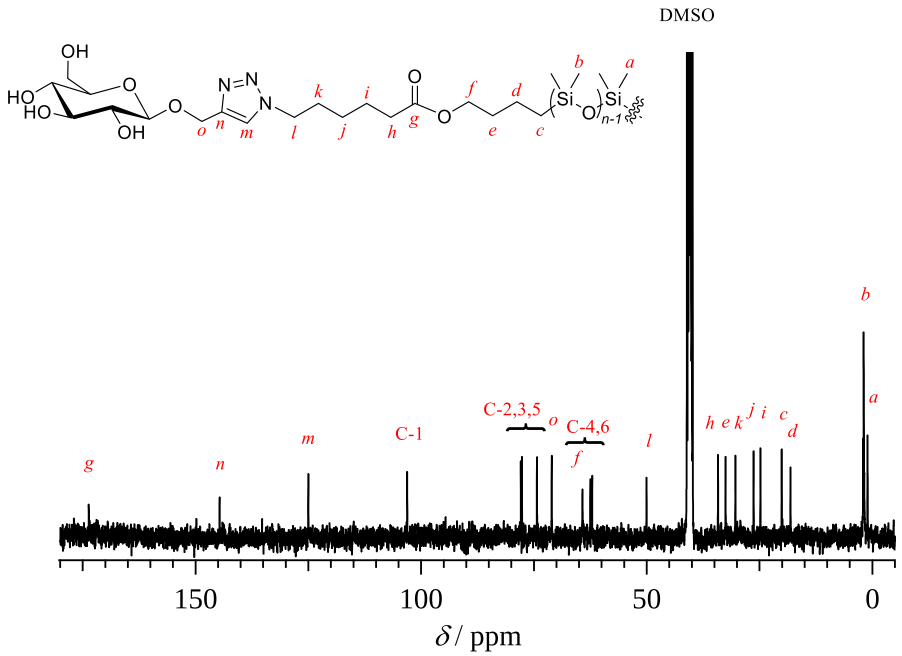

Figure S11. ^{13}C NMR spectrum of Glc$_1$-*b*-DMS$_n$-*b*-Glc$_1$ in $CDCl_3$ (100 MHz).

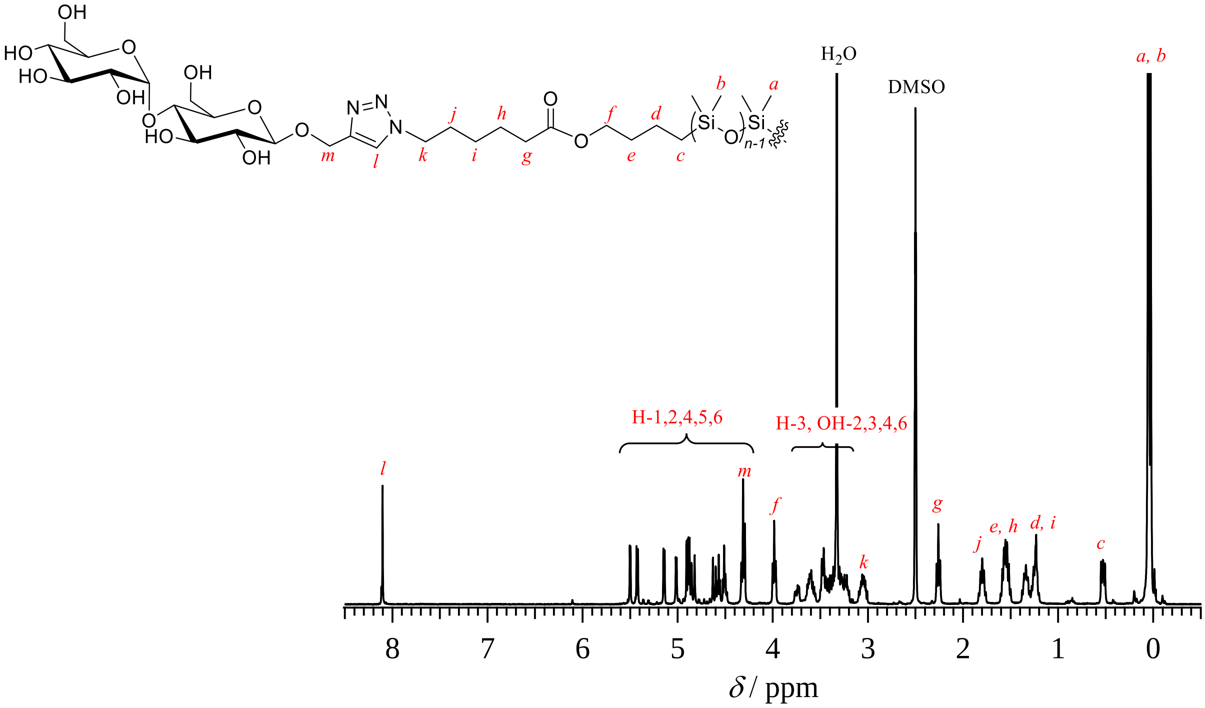

Figure S12. ^{1}H NMR spectrum of Glc$_2$-*b*-DMS$_n$-*b*-Glc$_2$ in $CDCl_3$ (400 MHz).

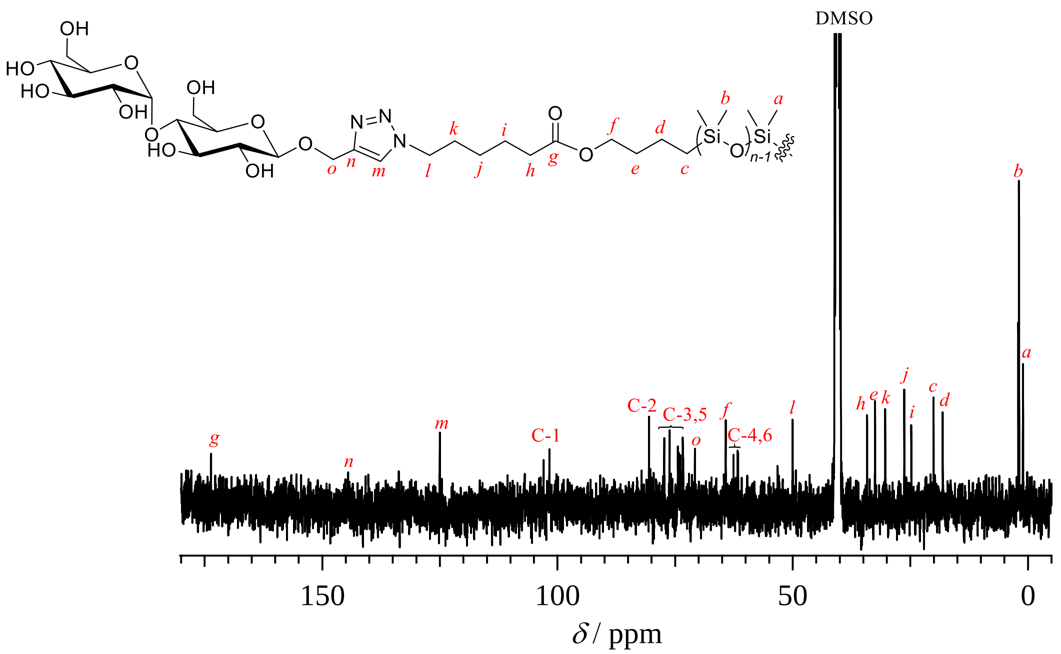

Figure S13. ^{13}C NMR spectrum of Glc$_2$-*b*-DMS$_n$-*b*-Glc$_2$ in $CDCl_3$ (100 MHz).

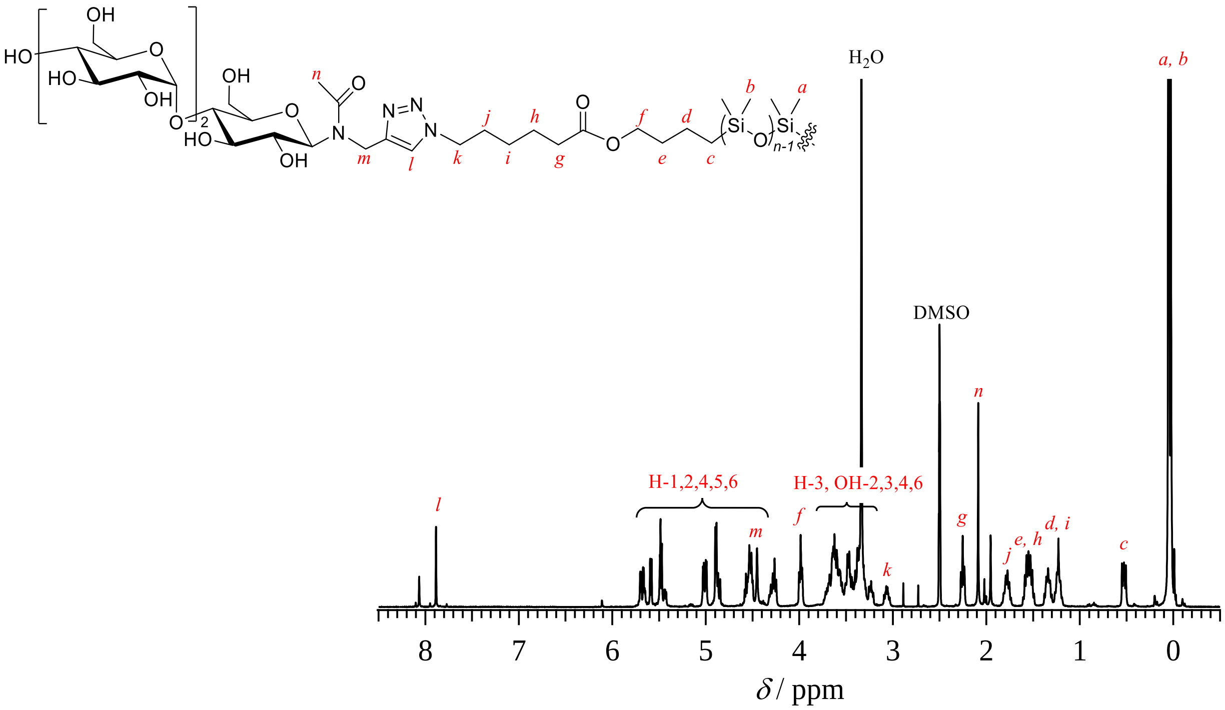

Figure S14. ^{1}H NMR spectrum of Glc$_3$-*b*-DMS$_n$-*b*-Glc$_3$ in $CDCl_3$ (400 MHz).

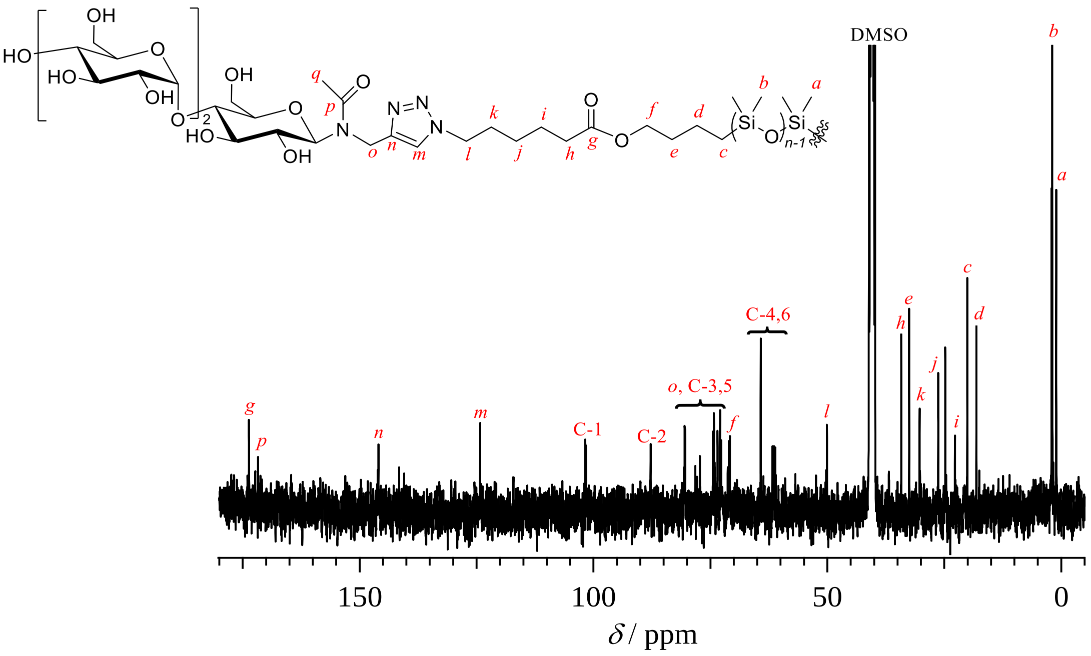

Figure S15. ^{13}C NMR spectrum of Glc$_3$-*b*-DMS$_n$-*b*-Glc$_3$ in CDCl$_3$ (100 MHz).

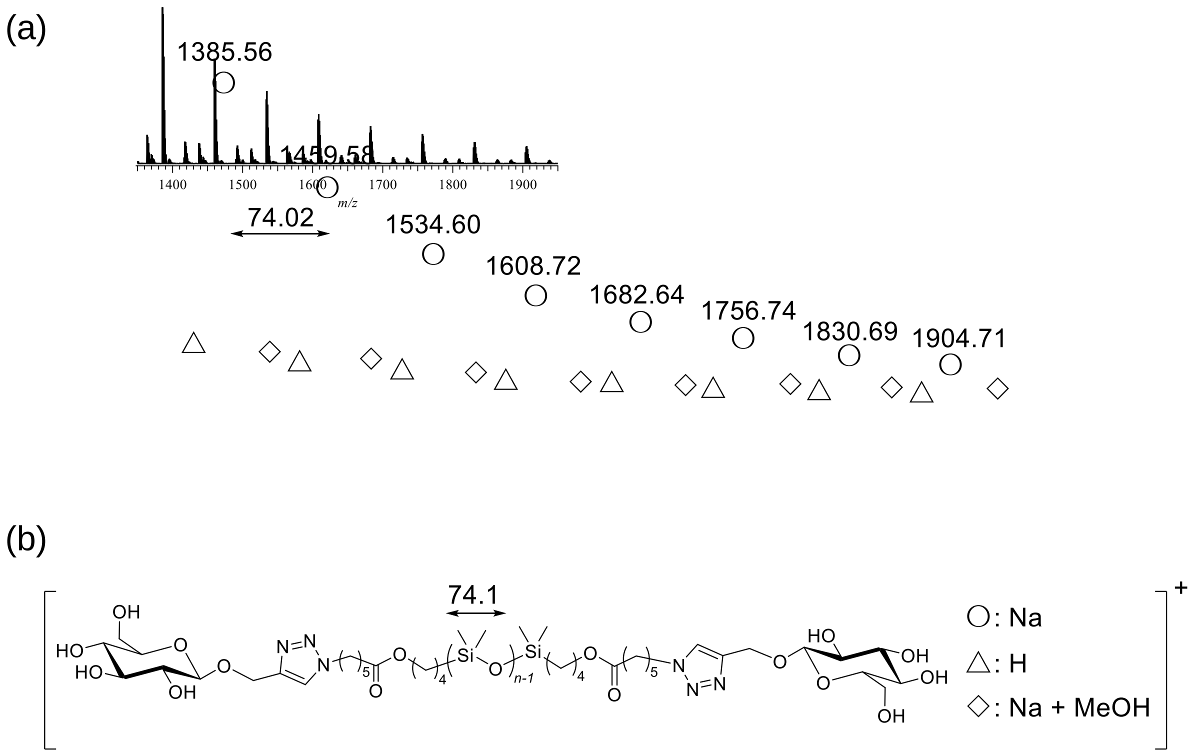

Theoretical molecular mass							
$n = 7$	$n = 8$	$n = 9$	$n = 10$	$n = 11$	$n = 12$	$n = 13$	$n = 14$
1385.56	1459.58	1533.60	1607.62	1681.64	1755.66	1829.68	1903.70

Figure S16. (**a**) ESI-MS spectrum of Glc$_1$-*b*-DMS$_n$-*b*-Glc$_1$ and (**b**) predicted molecular structures and theoretical molecular mass.

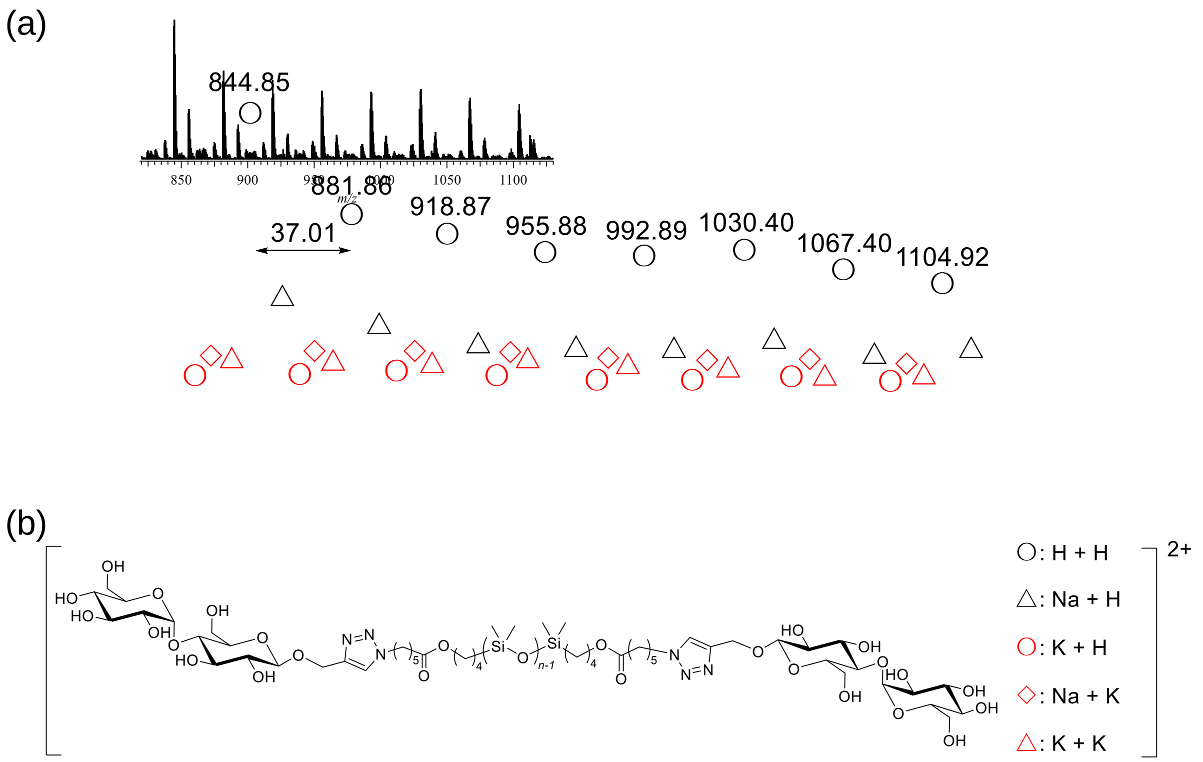

Theoretical molecular mass (z = 2)							
n = 7	n = 8	n = 9	n = 10	n = 11	n = 12	n = 13	n = 14
844.85	881.35	918.36	955.37	992.88	1029.89	1066.90	1103.91

Figure S17. (**a**) ESI-MS spectrum of Glc_2-*b*-DMS_n-*b*-Glc_2 and (**b**) predicted molecular structures and theoretical molecular mass.

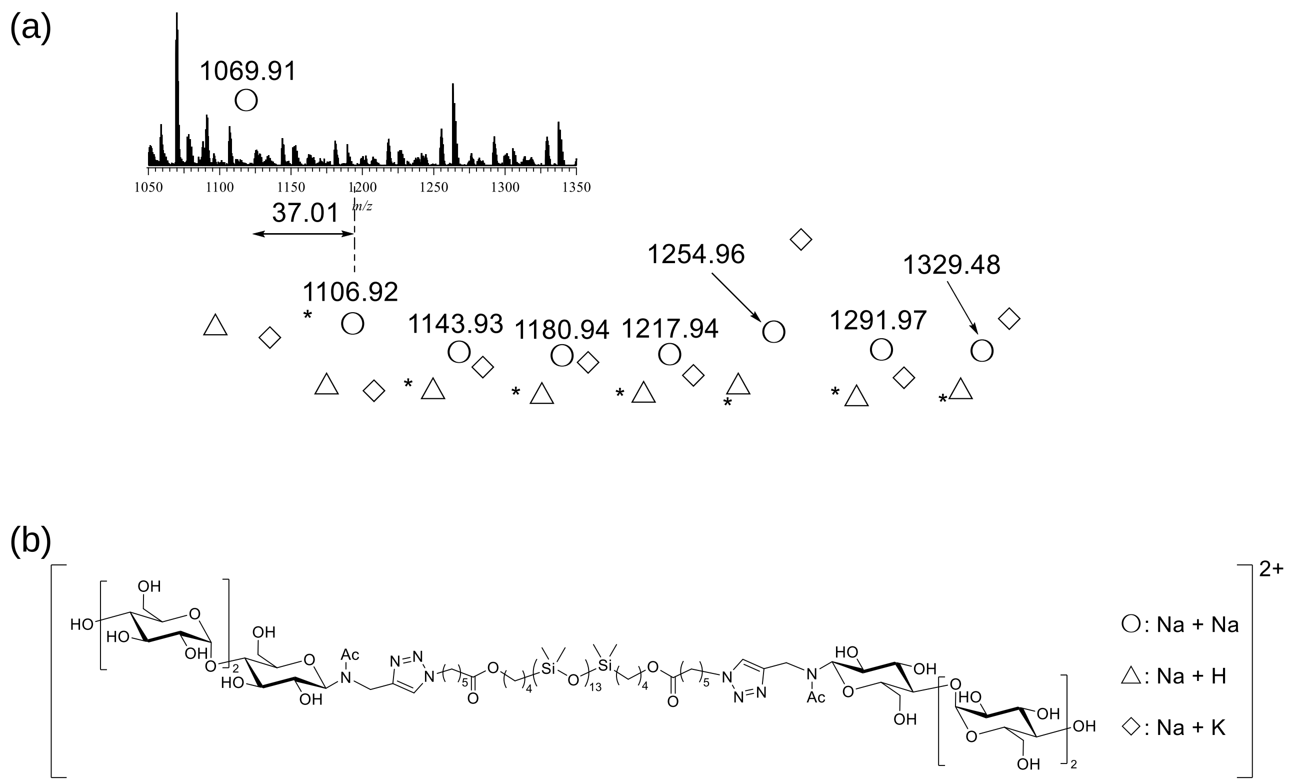

Theoretical molecular mass (z = 2)							
$n = 7$	$n = 8$	$n = 9$	$n = 10$	$n = 11$	$n = 12$	$n = 13$	$n = 14$
1069.91	1106.92	1143.93	1181.44	1218.45	1255.46	1291.96	1328.97

Figure S18. (**a**) ESI-MS spectrum of Glc_3-*b*-DMS_n-*b*-Glc_3 and (**b**) predicted molecular structures and theoretical molecular mass. * can't be attributed.

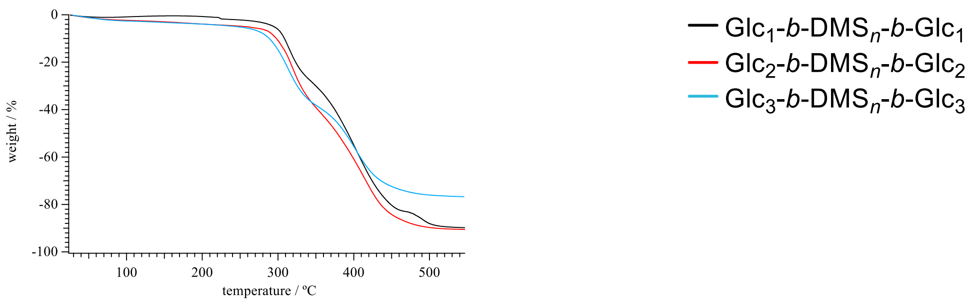

Figure S19. TGA curves of Glc1-*b*-DMS*n*-*b*-Glc1 (black), Glc2-*b*-DMS*n*-*b*-Glc2 (red) and Glc3-*b*-DMS*n*-*b*-Glc3 (blue).

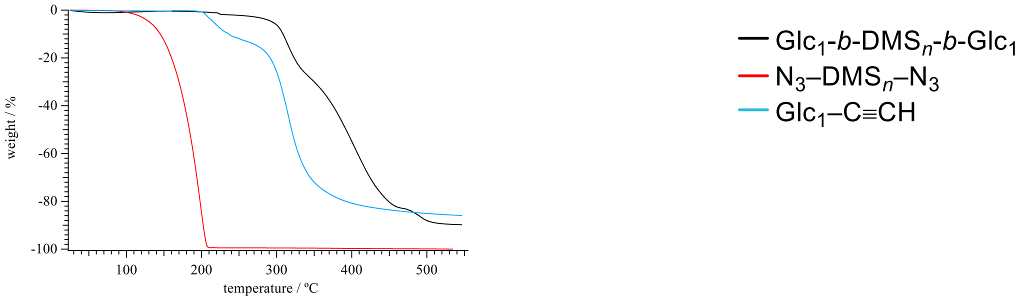

Figure S20. TGA curves of N3–DMS*n*–N3 (red), Glc1–C≡CH (blue), and Glc1-*b*-DMS*n*-*b*-Glc1 (black).

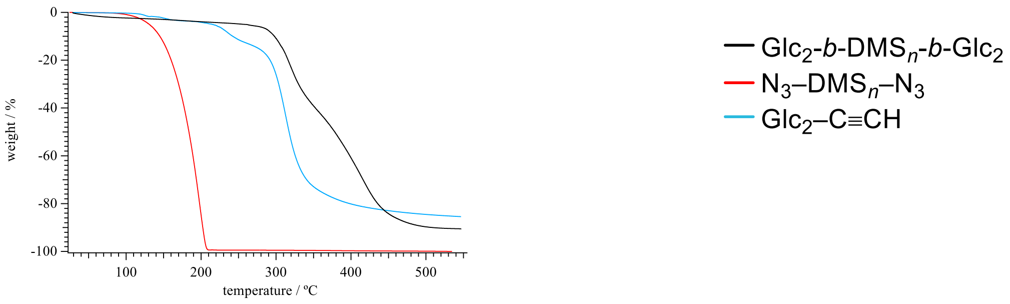

Figure S21. TGA curves of N$_3$–DMS$_n$–N$_3$ (red), Glc$_2$–C≡CH (blue), and Glc$_2$-*b*-DMS$_n$-*b*-Glc$_2$ (black).

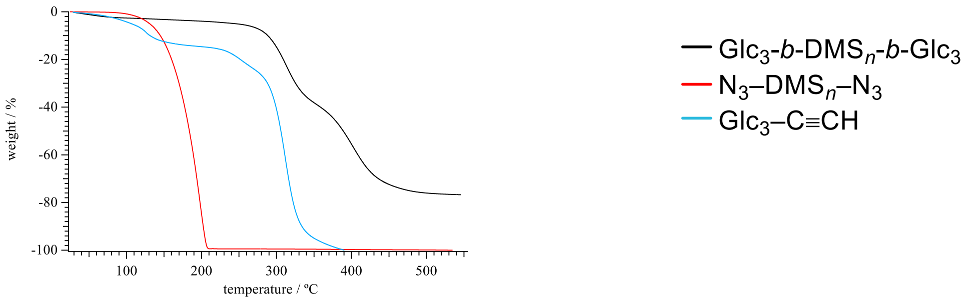

Figure S22. TGA curves of N$_3$–DMS$_n$–N$_3$ (red), Glc$_3$–C≡CH (blue), and Glc$_3$-*b*-DMS$_n$-*b*-Glc$_3$ (black).

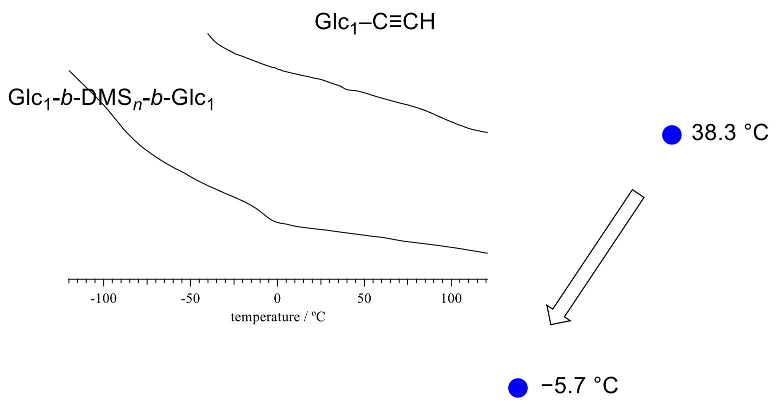

Figure S23. DSC curve of Glc$_1$-*b*-DMS$_n$-*b*-Glc$_1$.

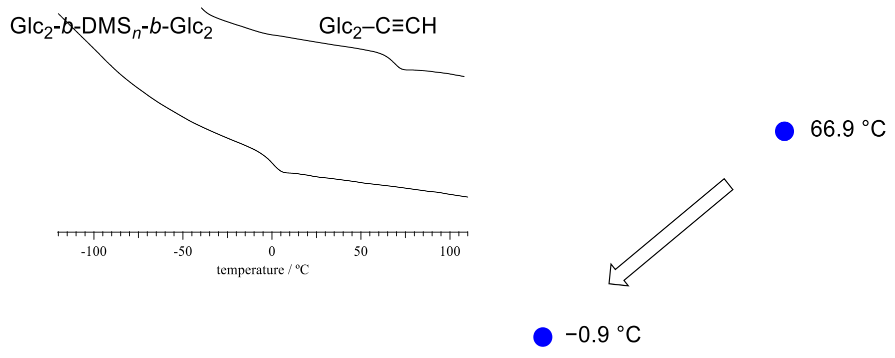

Figure S24. DSC curves of Glc$_2$-*b*-DMS$_n$-*b*-Glc$_2$.

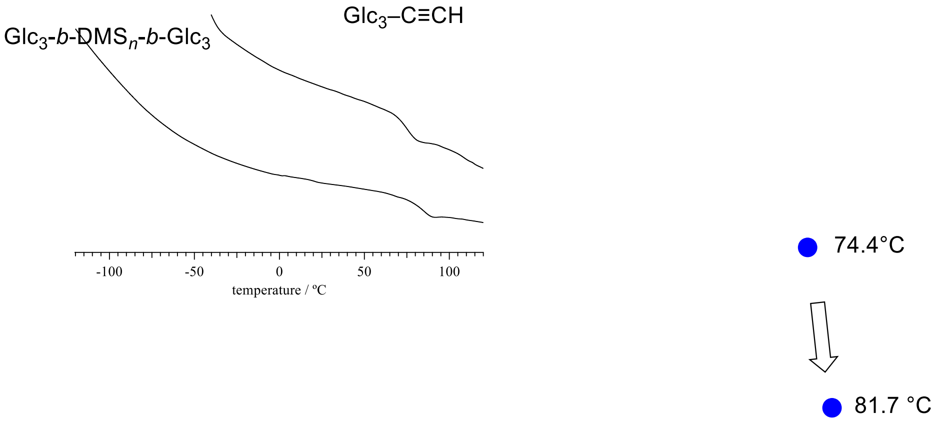

Figure S25. DSC curves of Glc_3-*b*-DMS_n-*b*-Glc_3.

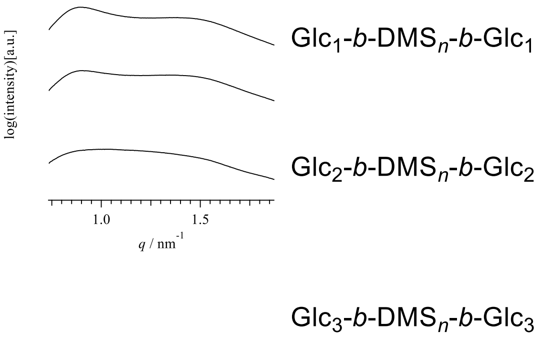

Figure S26. WAXS profiles of Glc_1-*b*-DMS_n-*b*-Glc_1 (upper), Glc_2-*b*-DMS_n-*b*-Glc_2 (middle), and Glc_3-*b*-DMS_n-*b*-Glc_3 (lower). All samples were annealed at 80 °C for 6 h.

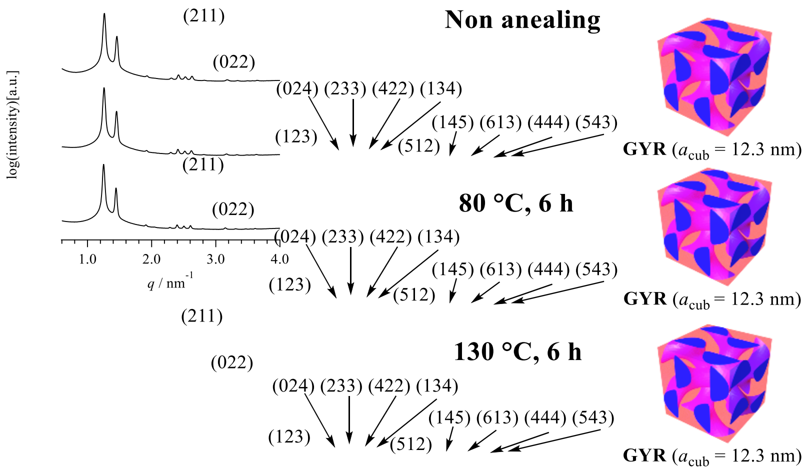

Figure S27. SAXS profiles of Glc$_1$-*b*-DMS$_n$-*b*-Glc$_1$ without annealing (upper), with annealing at 80 °C (middle), and 130 °C (lower) for 6h.

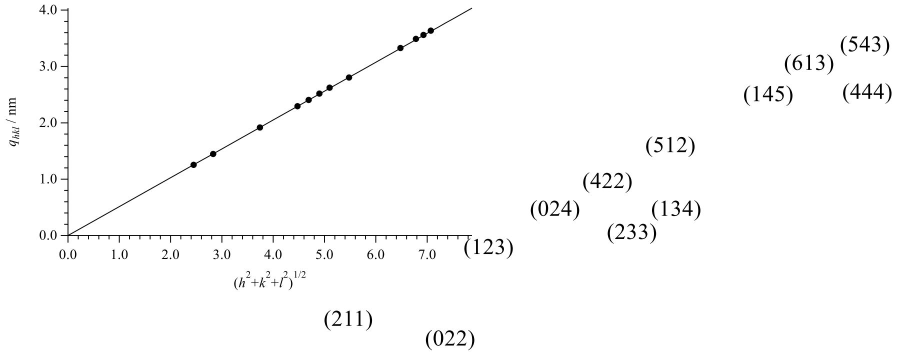

Figure S28. SAXS peak assignment of Glc$_1$-*b*-DMS$_n$-*b*-Glc$_1$ with annealing at 80 °C. The slope of a plot of q_{hkl} versus $(h^2 + k^2 + l^2)^{1/2}$ ($a_{cub} = 2\pi(h^2 + k^2 + l^2)^{1/2}/q_{hkl}$), where h, k, and l are Miller indices.

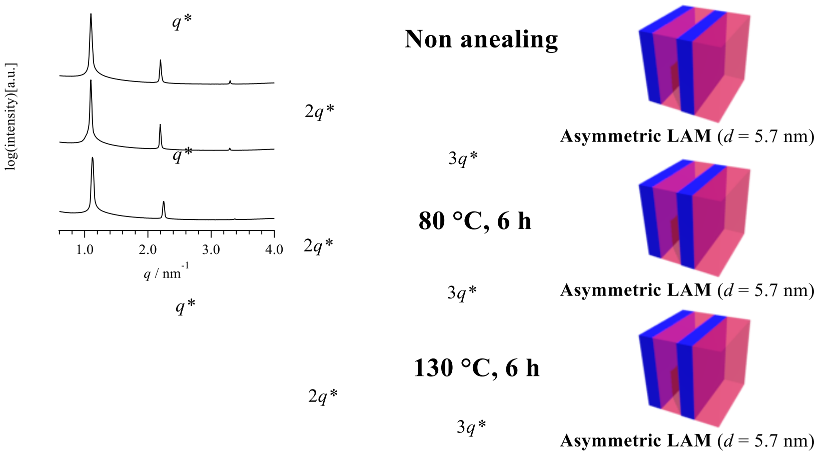

Figure S29. SAXS profiles of Glc_2-*b*-DMS_n-*b*-Glc_2 without annealing (upper), with annealing at 80 °C (middle), and 130 °C (lower) for 6h.

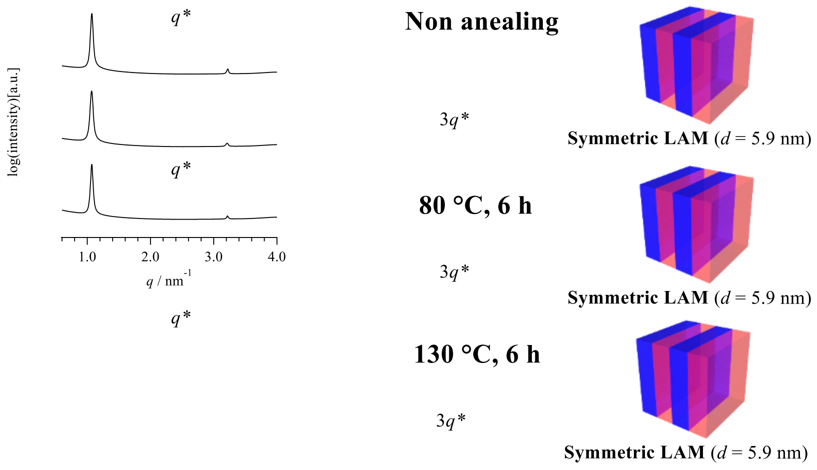

Figure S30. SAXS profiles of Glc_3-*b*-DMS_n-*b*-Glc_3 without annealing (upper), with annealing at 80 °C (middle), and 130 °C (lower) for 6h.

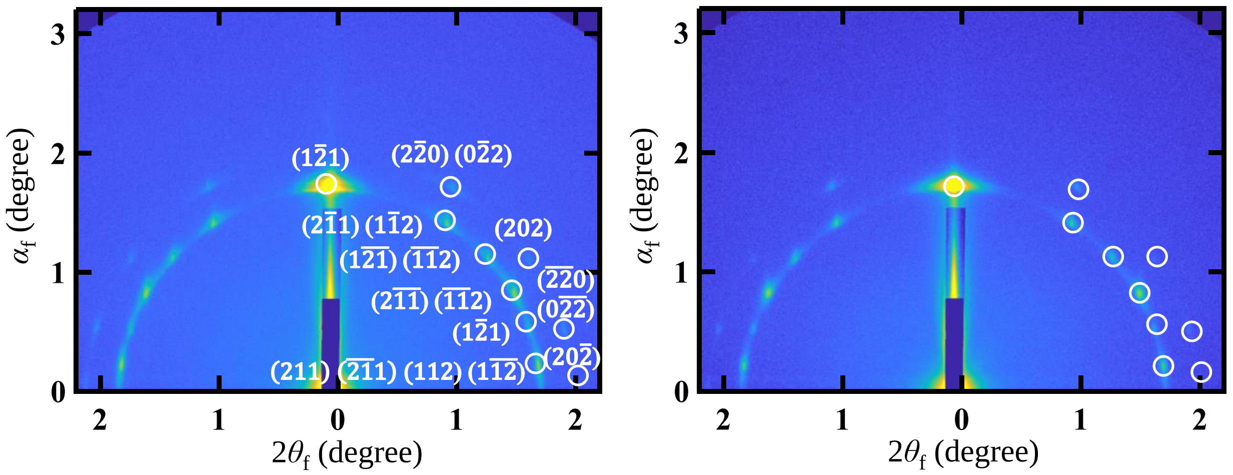

Figure S31. GISAXS images of Glc_1-*b*-DMS_n-*b*-Glc_1 thin films without annealing (left) and with annealing at 130 °C for 6h (right).

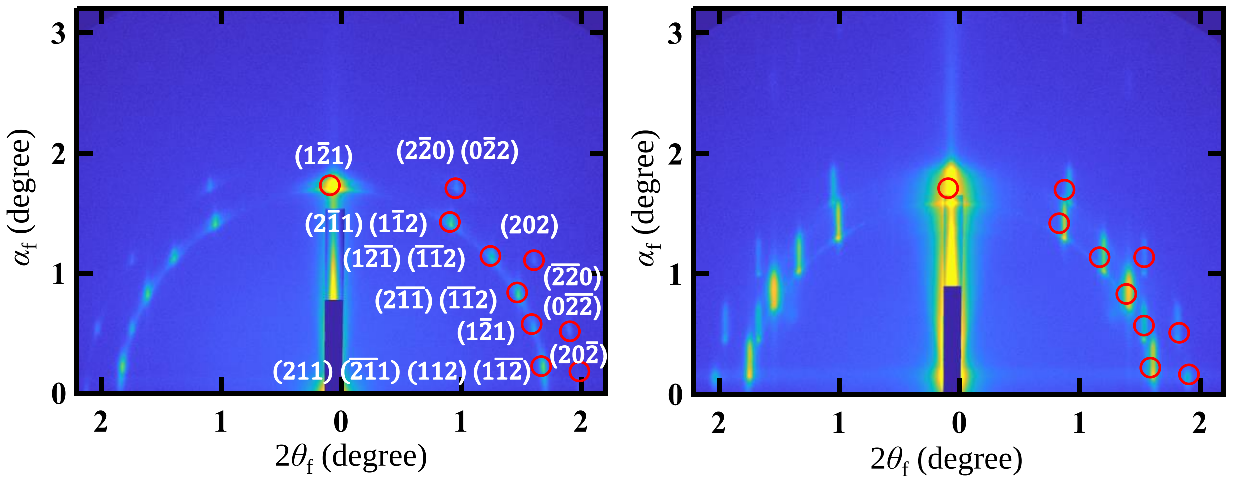

Figure S32. GISAXS image of Glc_1-*b*-DMS_n-*b*-Glc_1 thin films with annealing at 80 °C (left) and prepared eight months before (right).

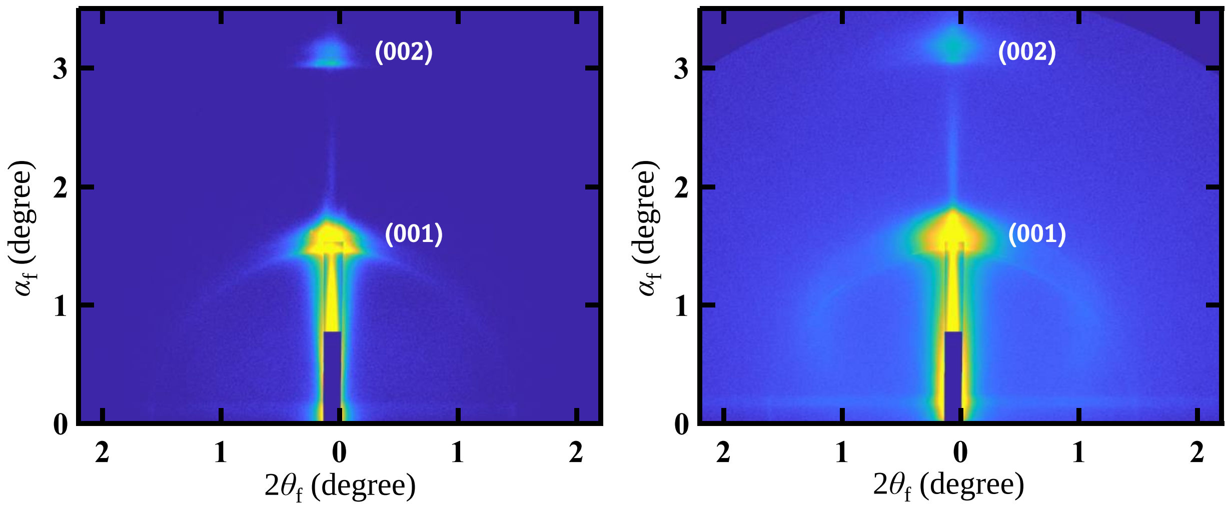

Figure S33. GISAXS images of Glc2-*b*-DMS*n*-*b*-Glc2 thin films without annealing (left) and with annealing at 130 °C for 6h (right).

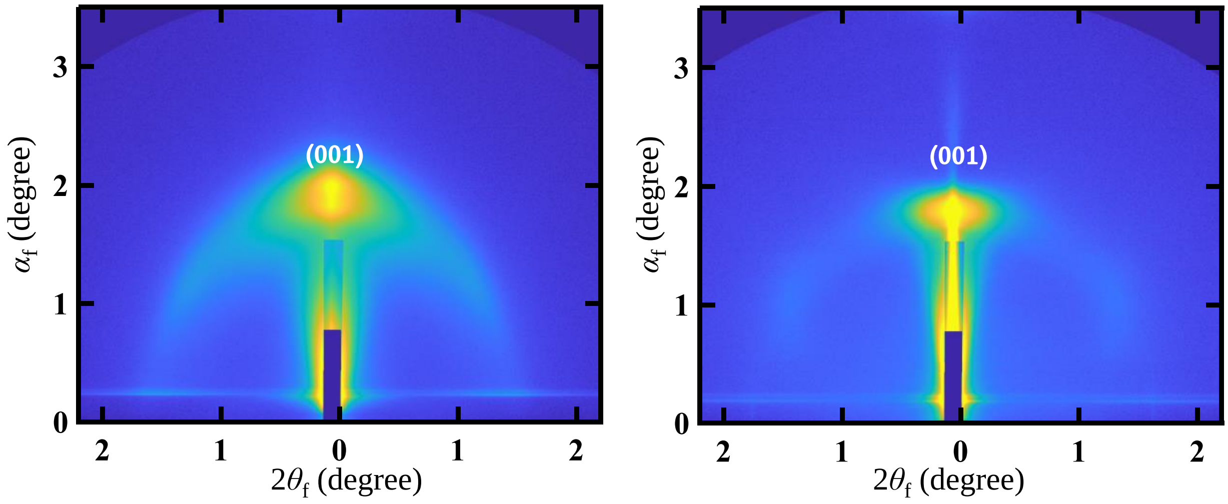

Figure S34. GISAXS images of Glc3-*b*-DMS*n*-*b*-Glc3 thin films without annealing (left) and with annealing at 130 °C for 6h (right).

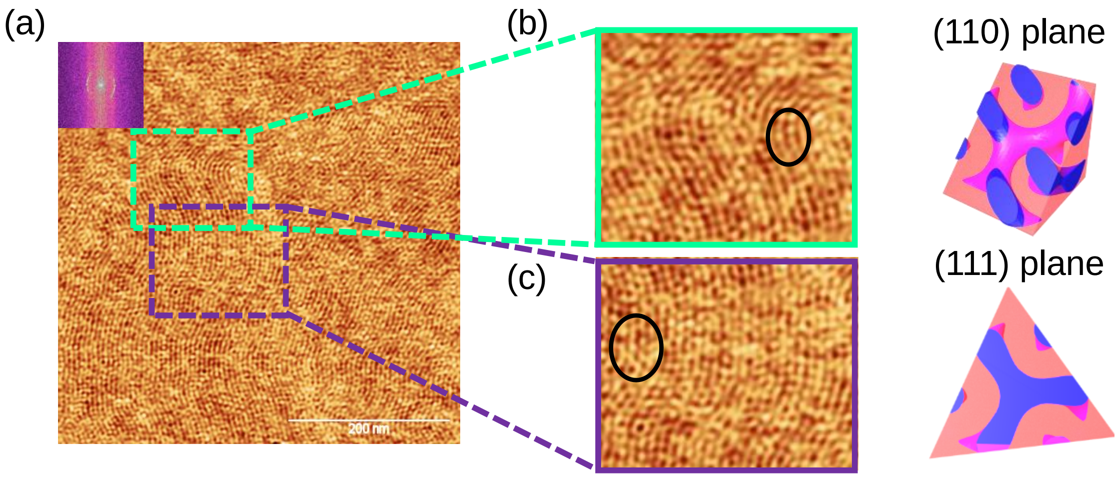

Figure S35. (**a**) AFM phase images of Glc$_1$-*b*-DMS$_n$-*b*-Glc$_1$ thin films with annealing at 80 °C for 6h. The insets show the FFT obtained from the phase images. (**b**) The image enlarged (110) plane microdomain from (**a**) (left) and the (110) plane image of GYR (right). (**c**) The image enlarged (111) plane microdomain from (**a**) (left) and the (111) plane image of GYR (right).

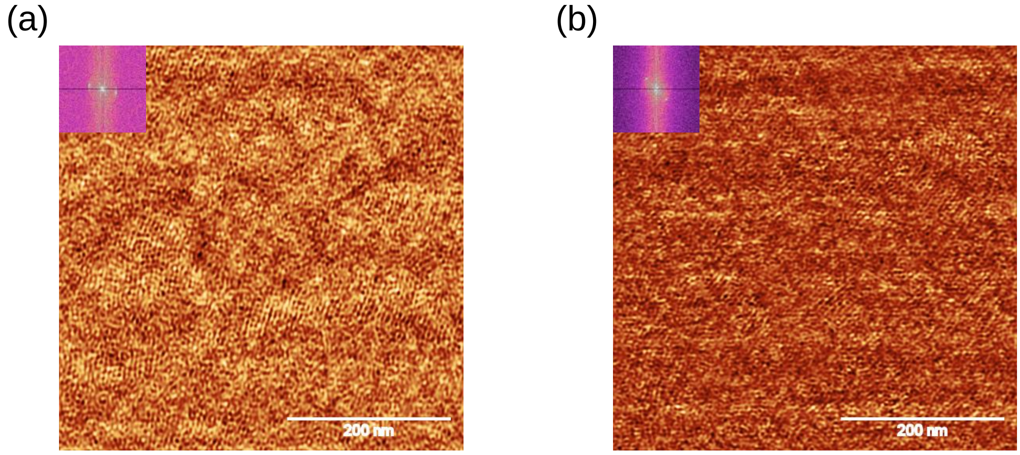

Figure S36. AFM phase images of Glc$_1$-*b*-DMS$_n$-*b*-Glc$_1$ thin films (**a**) without annealing and with annealing at (**b**) 130 °C for 6h. The insets show the FFT obtained from the phase images.

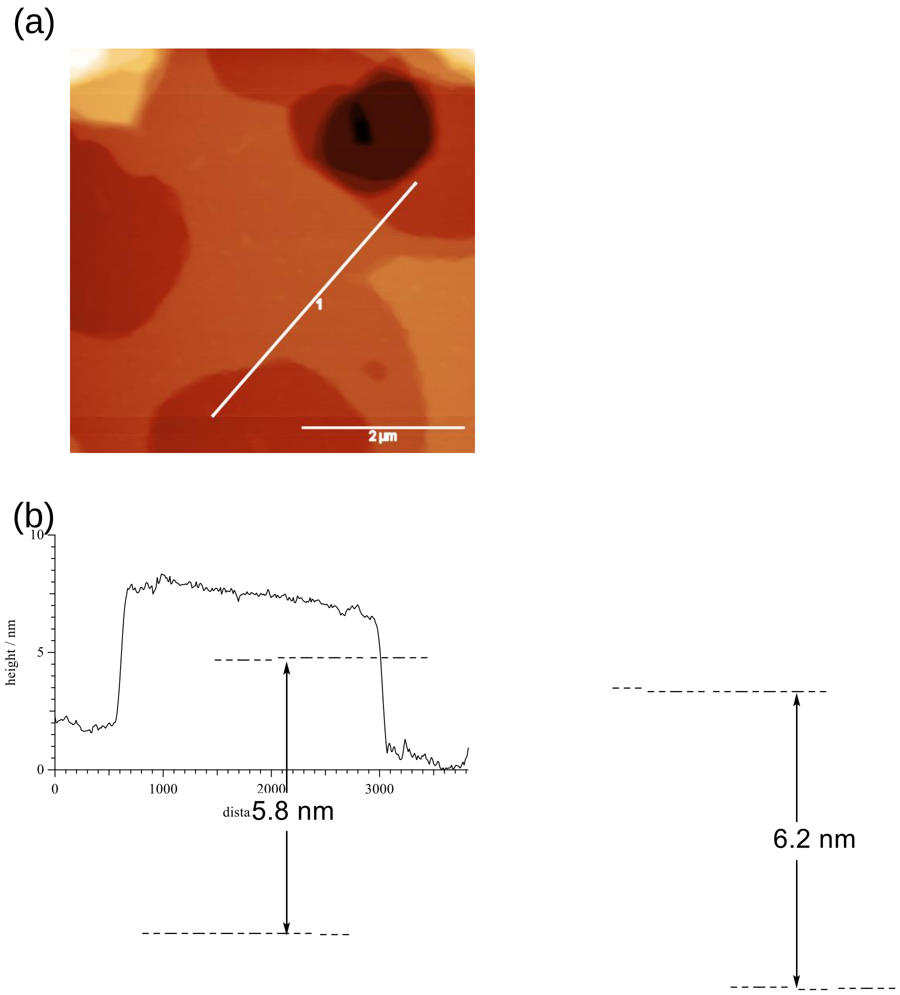

Figure S37. (**a**) AFM height images of Glc$_2$-*b*-DMS$_n$-*b*-Glc$_2$ thin films with annealing at 80 °C for 6 h. (**b**) AFM height cross sectional image along the white lines in (**b**).

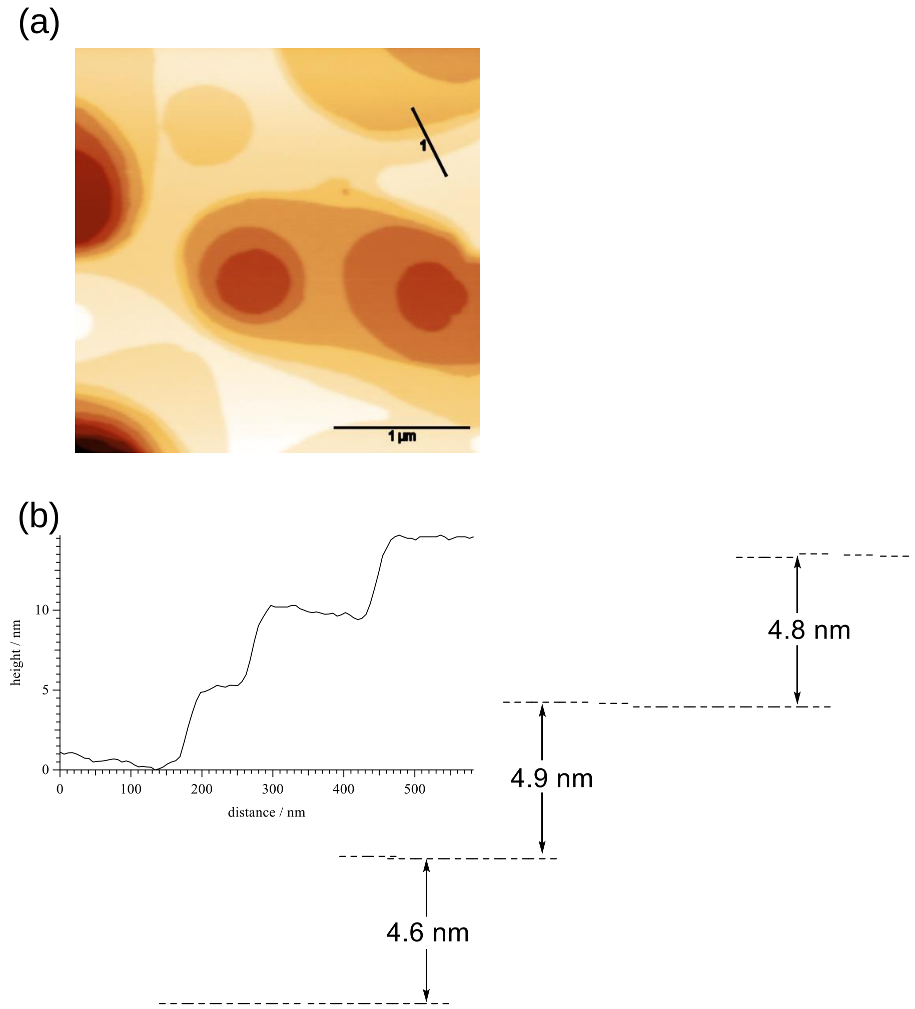

Figure S38. (**a**) AFM height images of Glc3-*b*-DMS*n*-*b*-Glc3 thin films with annealing at 130 °C for 6 h. (**b**) AFM height cross sectional image along the black lines in (a).

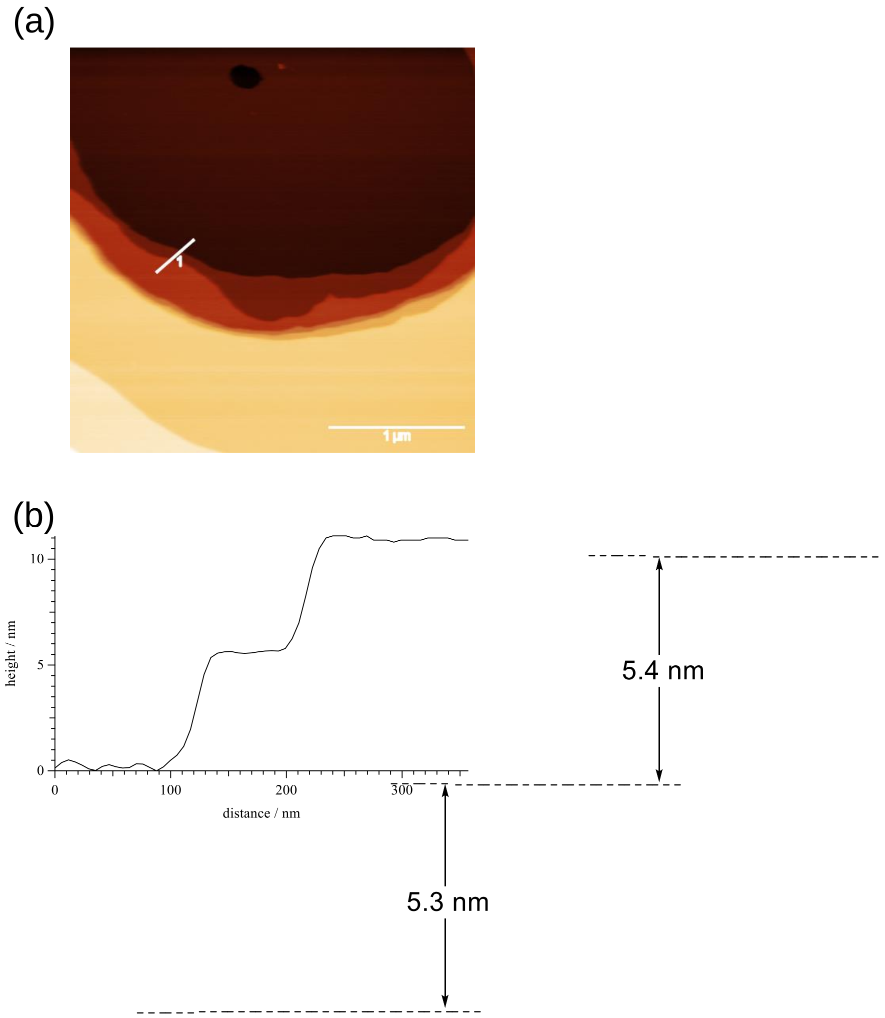

Figure S39. (**a**) AFM height images of Glc_2-*b*-DMS_n-*b*-Glc_2 thin films without annealing. (**b**) AFM height cross sectional image along the white lines in (**a**).

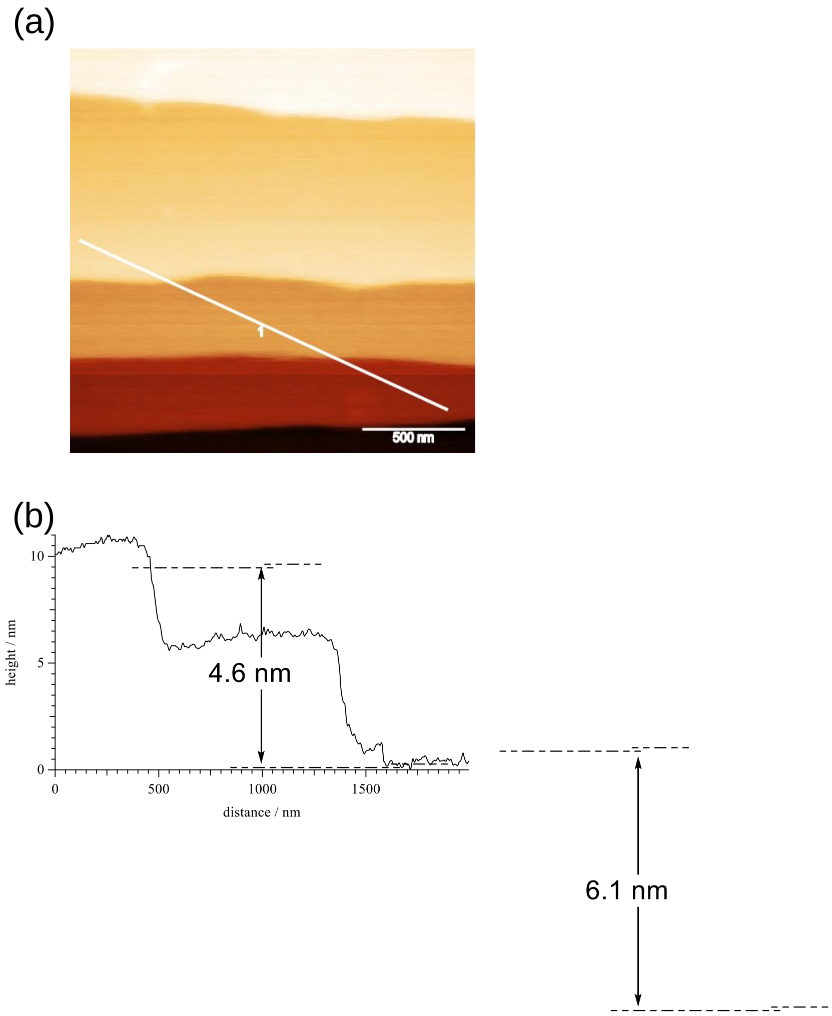

Figure S40. (**a**) AFM height images of Glc$_2$-*b*-DMS$_n$-*b*-Glc$_2$ thin films with annealing at 130 °C for 6 h. (**b**) AFM height cross sectional image along the white lines in (**a**).

(a)

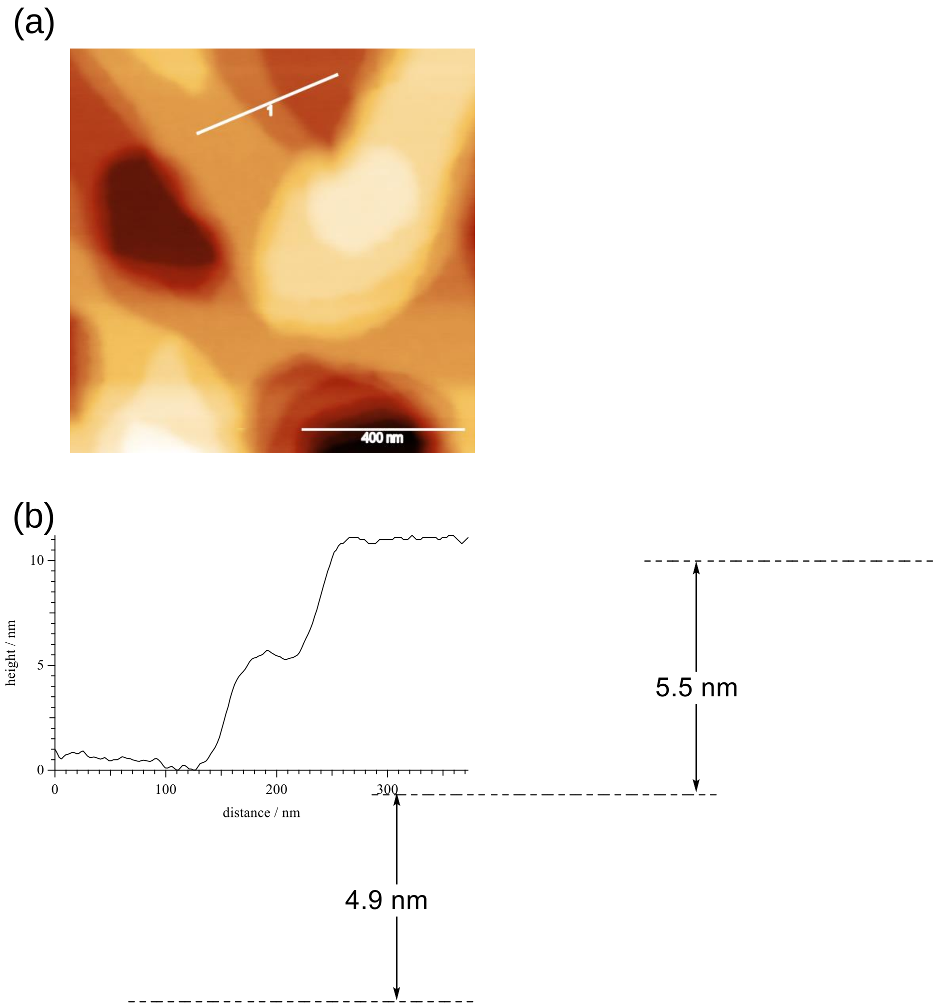

Figure S41. (**a**) AFM height images of Glc_3-*b*-DMS_n-*b*-Glc_3 thin films without annealing. (b) AFM height cross sectional image along the white lines in (**a**).

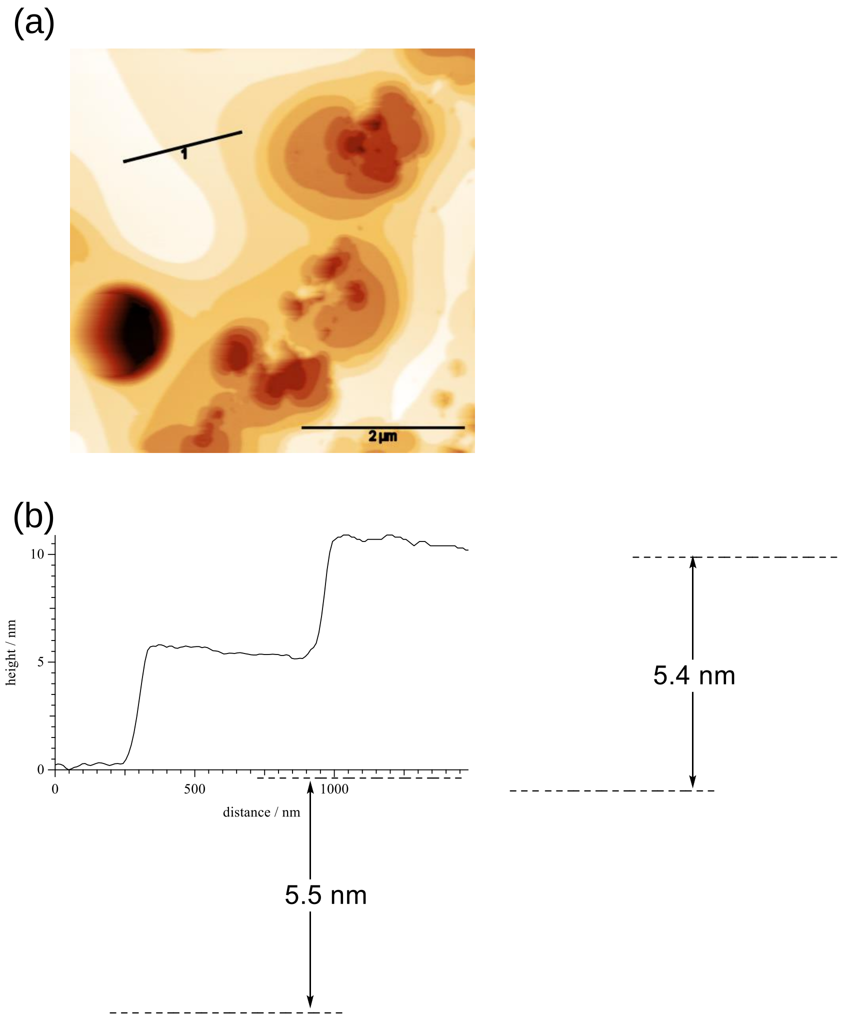

Figure S42. (**a**) AFM height images of Glc$_3$-*b*-DMS$_n$-*b*-Glc$_3$ thin films with annealing at 130 °C for 6 h. (**b**) AFM height cross sectional image along the black lines in (a).

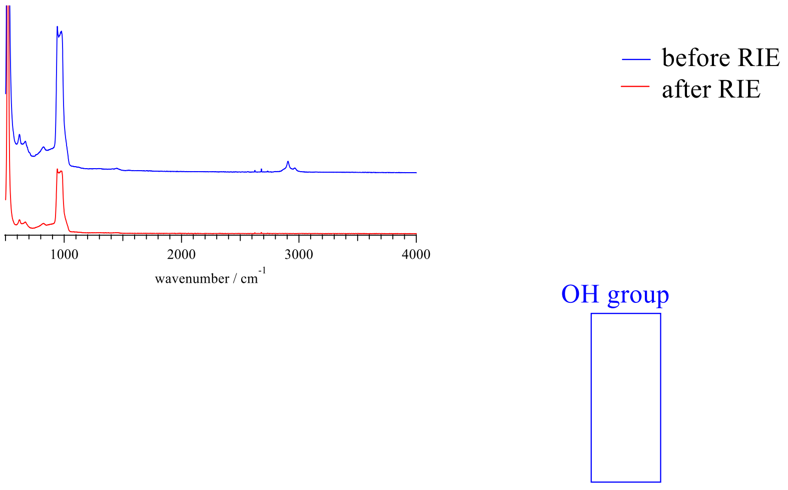

Figure S43. Raman spectra of Glc$_1$-*b*-DMS$_n$-*b*-Glc$_1$ in the thin film state before (upper) and after (lower) RIE.

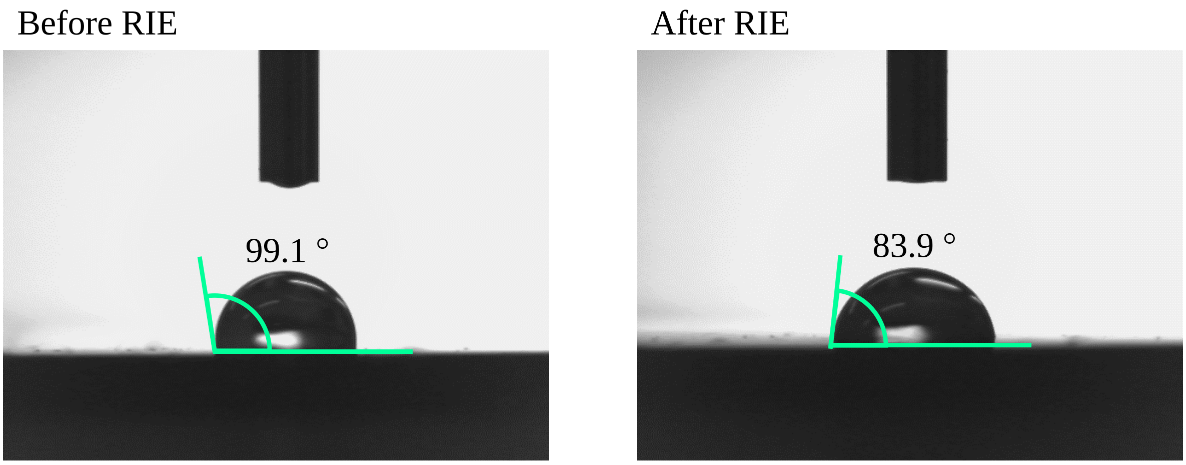

Figure S44. The water contact angle image of Glc$_1$-*b*-DMS$_n$-*b*-Glc$_1$ in the thin film state before (left) and after (right) RIE.

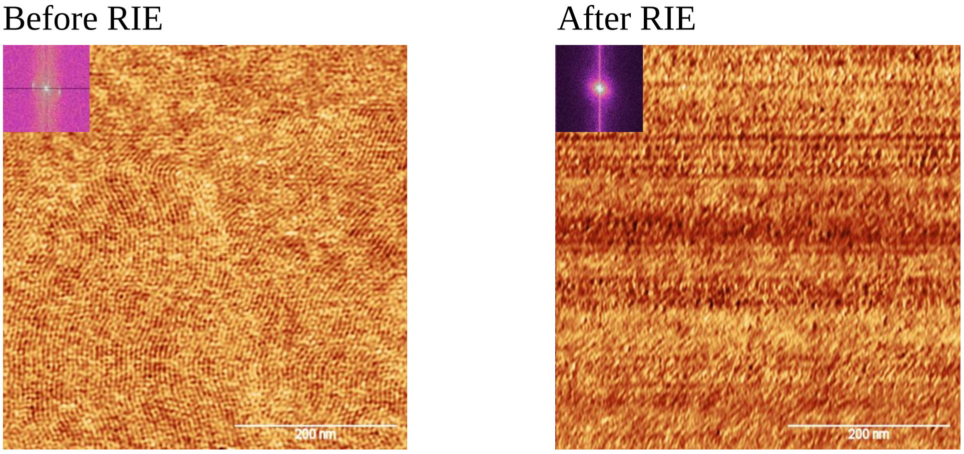

Figure S45. AFM phase images of Glc_1-*b*-DMS_n-*b*-Glc_1 thin films with annealing at 80 °C for 6h before (upper) and after (lower) RIE. The insets show the FFT obtained from the phase images.
